# Baseline AHR expression shapes immune response to pharmacological modulation in PBMCs from pancreatic cancer patients

**DOI:** 10.3389/fimmu.2025.1655258

**Published:** 2025-11-20

**Authors:** Arenida Bartkeviciene, Aldona Jasukaitiene, Inga Zievyte, Sandra Ivanauskiene, Gabija Stachneviciute, Kornelija Jenceviciute, Gabriele Karvelyte, Darius Stukas, Agne Sikarske, Daiva Urboniene, Toivo Maimets, Kristaps Jaudzems, Astra Vitkauskiene, Jason Matthews, Antanas Gulbinas, Zilvinas Dambrauskas

**Affiliations:** 1 Laboratory of Surgical Gastroenterology, Institute for Digestive Research, Lithuanian University of Health Sciences, Kaunas, Lithuania; 2 Department of Surgery, Medical Academy, Lithuanian University of Health Sciences, Kaunas, Lithuania; 3 Department of Laboratory Medicine, Lithuanian University of Health Sciences, Kaunas, Lithuania; 4 Department of Cell Biology, Institute of Molecular and Cell Biology, University of Tartu, Tartu, Estonia; 5 Department of Physical Organic Chemistry, Latvian Institute of Organic Synthesis, Riga, Latvia; 6 Department of Nutrition, Institute of Basic Medical Sciences, Faculty of Medicine, University of Oslo, Oslo, Norway; 7 Department of Pharmacology and Toxicology, University of Toronto, Toronto, ON, Canada

**Keywords:** AHR, PBMC, PDAC, immunotherapy, personalized medicine

## Abstract

**Background:**

Pancreatic ductal adenocarcinoma (PDAC) remains largely unresponsive to immunotherapy because of its highly immunosuppressive tumor microenvironment. Aryl hydrocarbon receptor (AHR), a ligand-dependent transcription factor, has emerged as a key regulator of immune homeostasis and inflammation. However, its systemic immunomodulatory role in PDAC, particularly outside the tumor microenvironment, remains poorly understood.

**Methods:**

Peripheral blood mononuclear cells (PBMCs) from patients with PDAC and healthy donors were isolated and treated *ex vivo* with two AHR agonists (Carbidopa and Tapinarof) and one antagonist (BAY 2416964). The samples were stratified into Low and High/Medium AHR expression groups. Flow cytometry (FC), qPCR, ELISA, Luminex assays, and immunofluorescence imaging were used to evaluate immune checkpoint expression, cytokine secretion, monocyte polarization, and subcellular AHR localization. Overall survival analysis was performed based on the baseline AHR expression levels.

**Results:**

Baseline AHR expression strongly influenced the immunological effects of AHR modulators. In High/Medium AHR PBMCs, Carbidopa increased PD-L1 and soluble PD-1 (sPD-1) levels, while IL10 expression was suppressed. In contrast, BAY significantly reduced PD-1 and sPD-1 levels in Low AHR PBMCs, whereas Tapinarof induced the highest IL10 expression. All modulators reduced the proportion of M2-like monocytes, indicating a shift toward less immunosuppressive phenotypes. Nuclear translocation of AHR protein varied across treatments and expression levels. Kaplan–Meier analysis revealed a non-significant trend toward improved overall survival in the High/Medium AHR group (log-rank p = 0.276).

**Conclusion:**

Baseline AHR expression critically shapes the immune response to pharmacological modulation in PBMCs from PDAC patients. These findings suggest that AHR profiling may serve as a clinically relevant biomarker for stratifying patients and guiding personalized immunotherapy approaches for PDAC.

## 1 Introduction

Pancreatic ductal adenocarcinoma (PDAC) is currently the third leading cause of cancer-related deaths, with a grim five-year survival rate of only about 10% (1, 2). The incidence of PDAC has been increasing, paralleling the growing prevalence of risk factors such as smoking, obesity, and diabetes (3). Although early detection can significantly improve survival rates, most patients with PDAC are diagnosed at an advanced stage, when metastasis has already occurred (4). Consequently, only 10–20% of patients are eligible for potentially curative surgery (5). Current standard treatments, including chemotherapy, immunotherapy, and radiotherapy, typically extend survival by weeks to months, highlighting the urgent need for novel therapeutic approaches (6).

Although immunotherapy has revolutionized treatment in many cancer types, its success in PDAC remains limited because of the tumor’s complex immune microenvironment and potent immunosuppressive mechanisms (7). PDAC tumors are highly heterogeneous and consist of malignant epithelial cells, fibroblasts, immune cells, and other stromal elements within a dense and reactive tumor microenvironment (TME) (2). This environment disrupts systemic immune homeostasis through reciprocal interactions between the immune cells and inflammatory cytokines. A deeper understanding of these immune evasion mechanisms is essential to improve immunotherapeutic outcomes (8).

Cytokines play crucial roles in orchestrating antitumor immunity; however, PDAC tumors frequently escape immune detection by downregulating antigen presentation pathways (9, 10). Recent therapeutic efforts have focused on targeting immune checkpoints, particularly the PD-1–PD-L1 axis (11). PD-L1 expression in tumors or immune cells can bind to PD-1 on T lymphocytes and suppress cytotoxic immune responses (12, 13). Unlike therapies that directly kill cancer cells, checkpoint inhibitors activate endogenous lymphocytes to elicit anti-tumor effects (11, 12). However, PDAC is often considered non-immunogenic, and clinical responses to checkpoint inhibitors remain poor, in part due to the presence of immunosuppressive populations such as regulatory T cells (Tregs) and tumor-associated macrophages (TAMs) (14).

TAMs are particularly influential in shaping the TME and are capable of switching from an M1 (tumoricidal) to an M2 (tumor-promoting) phenotype, thereby supporting immune evasion, angiogenesis, and metastasis (15, 16). While PD-1/PD-L1 inhibitors have shown efficacy in cancers such as melanoma and non-small cell lung cancer, PDAC patients demonstrate only a 10–30% response rate with rapid development of resistance (17, 18). Uncovering the mechanisms underlying this resistance is critical for improving therapeutic efficacy.

The aryl hydrocarbon receptor (AHR) has recently emerged as a pivotal mediator of inflammation and immune regulation in cancers including PDAC (19–24). Upon activation, AHR translocates to the nucleus and induces target genes, such as CYP1A1 and PTGS2 (COX-2), both of which are linked to tumor progression and immunosuppressive signaling. Elevated expression of AHR and its targets in the tumor microenvironment has been associated with cancer-driven immune evasion (25, 26).

Our previous study identified a marked reduction in AHR expression in peripheral blood mononuclear cells (PBMCs) from patients with more progressive PDAC, indicating systemic dysregulation of the AHR pathway beyond the tumor site (26).

Unlike murine models, which often fail to recapitulate the immunological complexity of human disease, *ex vivo* studies using patient-derived PBMCs offer translationally relevant insights into immune responses and therapeutic vulnerabilities. Human PBMCs preserve patient-specific immune signatures, cytokine profiles, and receptor expression patterns, which are essential for identifying biomarkers and tailoring immunomodulatory interventions.

We hypothesized that baseline AHR expression in PDAC patient-derived PBMCs dictates the magnitude and direction of immune response to AHR-targeted compounds. By stratifying patients based on their AHR status, we aimed to uncover personalized immunomodulatory strategies that could potentiate immune responsiveness in this otherwise immunologically refractory cancer type.

Motivated by these observations, the present study investigated how the pharmacological modulation of AHR affects immune checkpoint molecule expression, cytokine secretion, gene regulation, and monocyte polarization in PBMCs isolated from patients with PDAC.

## 2 Materials and methods

### 2.1 Patient population

**Figure 1:**
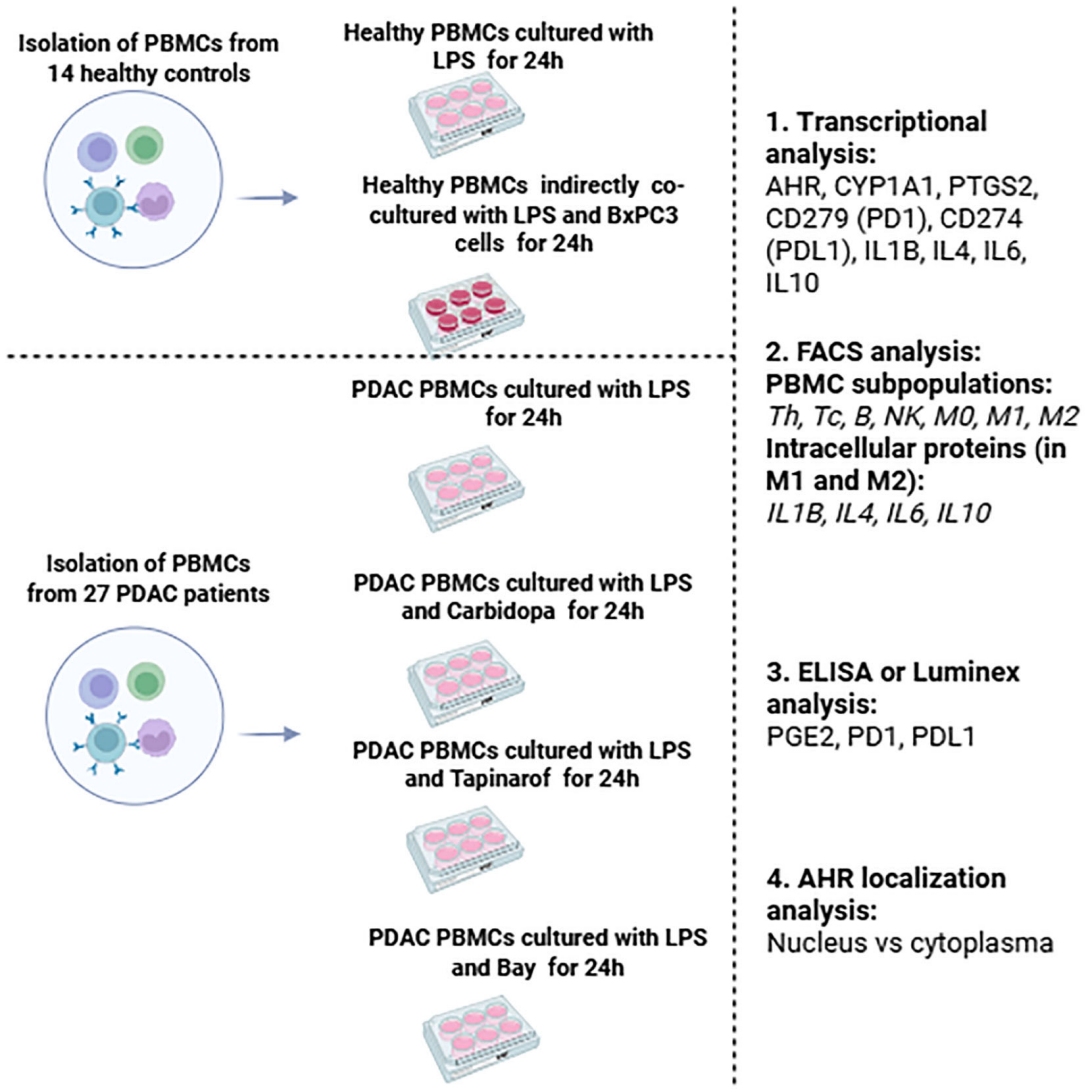
Schematic overview of the study design, including PBMC isolation from PDAC patients and controls: healthy PBMCs cultivation and indirect co-cultivation with BxPC-3 cells, PDAC PBMCs treatment with AHR modulators, and subsequent immunological analyses of all groups. The main criteria for this study group of cancer patients were as follows.

PBMCs were isolated from the venous blood of patients with PDAC and healthy controls (cultured healthy PBMCs and healthy PBMCs co-cultured with BxPC-3 cells; **Figure 1**). The PDAC patient group had a median age of 68 years (range: 54-80), consisted of 16 women and 11 men. The control group had a median age of 59 years (range: 43-69), comprised nine women and five men. A total of 27 PDAC patient samples were used, which were grouped according to the following modulators: Carbidopa (n=20), Tapinarof (n=19), and BAY (n=19). Samples could be assigned to multiple treatment groups, explaining the overlap in the numbers obtained from patients with PDAC whose pancreatic cancer was histologically confirmed, either during surgery or biopsy. For healthy control blood samples were collected from 14 healthy donors with no history of cancer. Negative control (CTRL) wells contained PDAC or healthy PBMCs were activated with lipopolysaccharides but without modulators or BxPC-3 cells. Because PBMC yield and viability varied between donors and assays, and because AHR stratification was performed *post hoc* based on baseline AHR mRNA levels, the number of evaluable samples (n) per treatment condition differed between AHR groups. When sufficient cell numbers were available, aliquots from the same donor contributed to multiple treatment conditions, which explains the partial overlap in n values across experimental arms. Detailed sample numbers per assay and condition are summarized in **Supplementary Table ST1**.

- Clinically, radiologically and cytologically/histologically diagnosed with pancreatic cancer.
- Surgical treatment of cancer was planned.
- The patient was treated at The Hospital of Lithuanian University of Health Sciences (LUHS) Kaunas Clinic.
- The age range was 20–90 years.
- The main criteria for the control group were as follows:
- No cancer was diagnosed clinically, radiologically or cytologically/histologically.
- Not currently suffering from a viral or bacterial infection shows no signs of an inflammatory disease (no fever, pain, fatigue, or weakness).
- The volunteer declared no use of any medications, including anti-inflammatory agents, analgesics, or antibiotics, within three days prior to the blood test.
- The age range was 20–90 years.

All samples were collected with informed consent from the patients, and the study protocol was approved by the Kaunas Regional Biomedical Research Ethics Committee (approval nr. BE-2-62).

### 2.2 PBMC isolation and cultivation

Peripheral blood collected in vacutainers containing EDTA K2 (BD Biosciences, Cat# 367525; Franklin Lakes, NJ, USA) was immediately centrifuged at 2500 g for 10 min at 20 °C for plasma separation. PBMCs were isolated using Ficoll-Paque PREMIUM gradient centrifugation (Cytiva, Cat# GE17-5442-03, Marlborough, MA, USA) according to the manufacturer’s protocol. PBMCs from patients and healthy individuals were subjected to repeated centrifugation and dilution. PBMC concentration was adjusted to 1 × 10^6^ cells/mL in RPMI 1640 medium (Gibco Life Technologies, Cat# 61870-044, Grand Island, NY, USA) supplemented with 10% fetal bovine serum (FBS; Thermo Scientific, Cat# 10500064, Waltham, MA, USA) and 1% penicillin/streptomycin (Gibco Life Technologies, Cat# 15140-122). The cell concentrations were determined using the trypan blue exclusion method (Carlsbad, Cat# 15250-061, CA, USA). PDAC PBMCs were seeded in 6-well plates (Corning, Cat# 3516; NY, USA) containing 1900 µL of complete RPMI medium at a density of 2 × 10^6^ cells/well. Three wells were seeded for each modulator and negative control. PBMC were activated with lipopolysaccharide (LPS; Sigma-Aldrich, Cat# L2630-25MG, St. Louis, MO, USA) at a final concentration of 0.5 µg/mL and incubated at 37 °C for 30 min. The same LPS concentration and incubation time were applied to all donor samples to ensure experimental consistency across treatment groups. The following modulators were used: Carbidopa (Sigma-Aldrich, Cat# 1095506, 410 µM), BAY 2416964 (Sigma-Aldrich, Cat# HY-135829, 400 nM), and Tapinarof (Thermo Scientific, Cat# HY-109044; Waltham, MA, USA, 80 µM). After stimulation, 100 µL of each modulator or vehicle control was added and the cells were incubated for 24 h at 37 °C. The cells were collected using a cell scraper (Corning, Cat# 3010) and centrifuged at 200×g for 10 min at 20 °C. The concentration was determined using trypan blue exclusion assay. A small portion of the pellets was analyzed immediately, while the remaining pellets and supernatants were stored at −80 °C. Healthy PBMCs were prepared 24 h after indirect co-cultivation with BxPC-3 cells (ATCC, Cat# CRL-1687, RRID: CVCL_0186, Manassas, VA, USA) obtained from the European Pancreas Center (Heidelberg, Germany). BxPC-3 cells were grown in RPMI medium in 25 cm² tissue culture flasks (Corning, Cat# 430639). Once confluent, the cell counts were determined using trypan blue. PBMCs were seeded at 2 × 10^6^ cells/well in 6-well plates, and 30 mm Millicell^®^ tissue culture inserts (Merck Millipore, Cat# PICM03050, Burlington, MA, USA) with 0.4 µm membranes were added. BxPC-3 cells were seeded onto the membrane at a density of 2 × 10^10^ cells/insert. After 24 h, the PBMCs were collected, centrifuged at 200×g, and stored as described above.

### 2.3 RNA extraction and real-time polymerase chain reaction

Total RNA was extracted from PBMCs using an RNA Extraction Kit (Abbexa, Cat# abx098089, Cambridge, UK) according to the manufacturer’s protocol. RNA purity and quantity were assessed using NanoDrop 2000 spectrophotometer (Thermo Fisher Scientific, Cat# B249; Waltham, MA, USA; RRID: SCR_018042). Complementary DNA was synthesized from 2 µg total RNA using a High-Capacity cDNA Reverse Transcription Kit (Applied Biosystems, Cat# 4368814, Waltham, MA, USA). The qPCR reactions (20 µL) included the synthesized cDNA, PCR master mix, and TaqMan Gene Expression Assays (Applied Biosystems) for AHR (Hs00169233_m1), IL1B (Hs00155410_m1), IL4 (Hs00174122_m1), IL6 (Hs00174131_m1), IL10 (Hs00961619_m1), CD279 (Hs05043241), CD274 (PD-L1, Hs00204257), PTGS2 (Hs00153133_m1), CYP1A1 (Hs01054796_g1), and GAPDH (Hs02786624). RT-PCR analysis was performed using ABI 7500 fast Real-Time PCR system (Applied Biosystem, Waltham, MA, USA).

### 2.4 Stratification of AHR expression groups

Patients were stratified into Low and High/Medium AHR expression groups based on baseline AHR mRNA levels. A fold-change cutoff of 0.5 relative to untreated PDAC PBMCs was used to define Low expression. This threshold corresponds closely to the 33rd percentile used in our previous publication (27), ensuring methodological consistency and reflecting biologically relevant divergence in immune phenotypes.

### 2.5 Flow cytometry (lymphocyte subsets, monocyte/macrophage subsets)

Flow cytometric immunophenotyping was performed using a BD Multitest™ 6-color T, B, NK cell panel (TBNK) Kit (BD, Cat# 337181, Franklin Lakes, NJ, USA) to determine the following lymphocyte subsets: CD19^+^ (B lymphocytes), CD3^+^ (T lymphocytes), CD3^+^CD4^+^ (T helpers), CD3^+^CD8 + (T cytotoxic lymphocytes), and CD3^−^CD16^+^CD56^+^ (natural killer (NK) cells). Aliquots of 1 × 10^6^ PBMCs were incubated for 30 min at room temperature with a combination of fluorochrome-conjugated monoclonal antibodies (mAbs) targeting CD3 (FITC, Leu-4), CD4 (PE-Cy7, Leu-3a), CD8 (APC-Cy7, Leu-2a), CD16 (PE, Leu-1 lc), CD19 (APC, Leu-12), CD56 (PE, Leu-19), and CD45 (PerCP-Cy5.5, 2D1) (BD Biosciences, USA). After incubation, cells were washed and analyzed using a FACSLyric™ 10-color flow cytometer (BD Biosciences, San Jose, CA, USA). Up to 30,000 events were recorded per sample. Lymphocyte populations were gated based on side scatter (SSC) and CD45 expression. The percentage of antigen-positive cells within the lymphocyte gate was calculated.

Phenotypic analysis of monocyte/macrophage subsets was performed using mAbs against CD3 (PE, UCHT1, BD, Cat# 555333, RRID: AB_395740), CD14 (BV510, MϕP9, Cat# 563079, RRID: AB_2737993), CD16 (APC, B73.1, Cat# 561304, RRID: AB_10714780), CD80 (APC-H7, L307.4, Cat# 561134, RRID: AB_10565974), CD86 (PE-Cy7, 2331 (FUN-1), Cat# 561128, RRID: AB_10563077), CD163 (BV605, GHI/61, Cat# 745091, RRID: AB_2742705), CD206 (FITC, 19.2, Cat# 551135, RRID: AB_394065), HLA-DR (PerCP, L243, Cat# 347402, RRID: AB_2868847), CD19 (PE, HIB19, Cat# 555413, RRID: AB_395813), CD56 (PE, Cat# 555516, RRID: AB_395906), and CD66b (PE, G10F5, Cat# 561650, RRID: AB_10894591) (all from BD Biosciences, San Jose, CA, USA).

The following mAbs were used to assess intracellular cytokine production: anti-IL-1β (Pacific Blue, H1b-98, BioLegend, Cat# 511710, RRID: AB_2124350), anti-IL4 (MP4-25D2, BD, Cat# 564110, RRID: AB_2738599), anti-IL6 (MQ2-13A5, Cat# 563279, RRID: AB_2738113), and anti-IL10 (BV421, JES3-19F1, Cat# 567012, RRID: AB_2870004). Intracytoplasmic assessment of cytokines was done using the BD Cytofix/Cytoperm™ Plus Fixation/Permeabilization Kit (BD, Cat# 554715), and BD GolgiStop™ protein transport inhibitor (containing monensin), by adding 1 µL to 1 × 10^6^ PBMCs after 4 h of treatment. Surface and intracellular staining was performed according to the manufacturer’s protocol. The PMT voltages and compensation settings were adjusted using a BD CompBeads Anti-Mouse Ig κ/Negative Control Compensation Particle Set (BD Biosciences, Cat# 552843, San Jose, CA, USA; RRID: SCR_008926). For analysis of monocyte/macrophage subsets and cytokine production, up to 30,000 events were collected per sample.

Monocytes were initially identified as CD14^+^HLA-DR^+^ events after exclusion of debris (FSC/SSC), doublets (FSC-A/FSC-H), and non-monocytic populations (CD3^+^, CD19^+^, CD56^+^, CD66b^+^). Subsequent gating on CD14 vs. CD16 distinguished the following subsets:

- Classical monocytes: CD14^++^CD16^−^
- Intermediate (M2-type) monocytes: CD14^++^CD16^+^; CD206^high; CD163^+^; IL-6^+^; IL-4^+^; IL-10^+^
- Non-classical (M1-type) monocytes: CD14^+^CD16^++^; CD206^low; CD80^+^; CD86^+^; IL-12^+^; TNF^+^; IL-1^+^
- Other monocytes: CD14^+^CD16^−^ (undefined phenotype)

The M0 macrophage subset was defined as CD14^+^HLA-DR^+^, M1 as CD80^+^CD86^+^, and M2 as CD163^+^CD206^+^. Monocyte polarization states were assessed using multiparameter (10-color) flow cytometry as the percentage of cells within their respective gates. Representative gating examples and quality control plots are shown in **Supplementary Figure SF4**. Cytokine expression was assessed as the mean fluorescence intensity (MFI), which was further converted into percentages for comparison across conditions.

### 2.6 Luminex (concentrations of media cytokines) and ELISA (concentrations of PD-1, PD-L1, PGE2, and IL6)

The concentrations of cytokines IL-1β, IL4, and IL10 were quantified in the culture media collected after 24 h of healthy and PDAC PBMCs incubation using magnetic bead–based multiplex assays (Human Cytokine Premixed Multi-Analyte Kit, R&D Systems, Minneapolis, MN, USA) and analyzed using a Luminex 100 analyzer (Luminex Corporation, Cat# L100-XPONENT, Austin, TX, USA; RRID: SCR_018025). Cell culture supernatants were centrifuged at 16,000×g for 4 min at 4 °C to remove debris or precipitate and then processed according to the manufacturer’s protocol. Briefly, analyte-specific antibodies were pre-coated onto magnetic microparticles, each containing a unique fluorophore signature. Standards and samples were incubated with the beads, followed by biotinylated detection antibodies and streptavidin–phycoerythrin (SAPE). The unbound components were removed from the final washes and the beads were analyzed via dual-laser detection. The identity of each analyte was determined using the bead region and the corresponding signal intensity (MFI) was used to interpolate the cytokine concentrations from the standard curves. For ELISA, the culture media collected after 24 h of PBMC incubation was used to determine the concentrations of PD-1 (Abcam, Cat# ab252360, Cambridge, UK), PD-L1 (Abcam, Cat# ab277712, Cambridge, UK), PGE2 (Elabscience, Cat# E-EL-0034, Houston, TX, USA), and IL6 (Invitrogen, Cat# KHC0061, Carlsbad, CA, USA). The supernatants were thawed and clarified by centrifugation before analysis. Assays were performed according to the manufacturer’s instructions. All measurements were performed in technical triplicates to ensure reproducibility and accuracy. The ELISA was conducted using a Tecan Sunrise microplate reader (Tecan Group Ltd., Männedorf, Switzerland). Standard curves were generated and protein concentrations were calculated by interpolating the sample optical density (OD) values from the calibration curves.

### 2.7 Immunofluorescence and quantitative image analysis of AHR localization

After 24 h of PDAC PBMC modulation, cells were gently collected using a cell scraper (ROTH, Cat# EKX9.1, Karlsruhe, Germany) and centrifuged at 200×g for 10 min at 20 °C. The cell concentrations were determined using the trypan blue exclusion method (Sigma-Aldrich, Cat# 15250-061, CA, USA). A total of 2 × 10^10^ cells per well were seeded into 24-well tissue culture plates (TPP, Cat# 92424; Trasadingen, Switzerland) and centrifuged at 120×g for 10 min at 20 °C. The supernatant was removed and cells were fixed with 100% methanol (Sigma-Aldrich, Cat# 1ET5.2, St. Louis, MO, USA) for 30 s, followed by three washes with PBS (Thermo Fisher Scientific, Cat# 10010023, Waltham, MA, USA). Permeabilization was performed using 0.1% Triton X-100 (Sigma-Aldrich, Cat# X100-100ML) in PBS for 10 minutes and then blocked with 3% bovine serum albumin (BSA; Cat# A-9647-100G; Sigma-Aldrich, St. Louis, MO, USA) in PBS for 1 h at room temperature. The cells were incubated with monoclonal mouse anti-AHR primary antibody (Thermo Scientific, Cat# MA1-514, Waltham, MA, USA) diluted 1:2000, followed by Alexa Fluor^®^ 488-conjugated goat anti-mouse IgG (H+L) secondary antibody (Invitrogen, Cat# A11001, Carlsbad, CA, USA) diluted 1:1000. All antibody incubations were followed by washing with PBS to remove the unbound reagents. For nuclear staining, the cells were incubated with Hoechst 33342 (for DAPI) (Thermo Scientific, Cat# H3570, Waltham, MA, USA) at 1 µg/mL in PBS for 10 min, followed by three additional PBS washes. Fluorescence imaging was performed in PBS by using an IX71 inverted fluorescence microscope (Olympus Corporation, Tokyo, Japan). The experiment was performed in triplicate, and a representative replicate is presented. To quantify AHR subcellular localization, custom Python-based scripts were used to segment the cell and nuclear regions based on phase-contrast and 4′,6-diamidino-2-phenylindole (DAPI) images, respectively. Cell boundaries were identified using Sobel filtering and adaptive Otsu thresholding of phase-contrast images, whereas nuclear segmentation was refined using watershed transformation of Hoechst-stained nuclei. The cytoplasmic compartment was determined by subtracting the nuclear mask from total cell mask. The AHR fluorescence intensity was calculated separately in the nuclear and cytoplasmic regions, and nuclear-to-cytoplasmic (N/C) ratios were determined per image. Representative images include the overlaid quantification results.

### 2.8 Statistical analysis

Statistical analyses were performed using GraphPad Prism (version 9.01; GraphPad Software Inc., San Diego, CA, USA; RRID: SCR_002798) and Python (version 3.11) with lifelines and matplotlib libraries. Data are presented as medians and are visualized using box plots, with the minimum and maximum values indicated. Group comparisons were performed using the nonparametric two-tailed Mann–Whitney U test. Statistical significance was set at p < 0.05.

Associations between clinical and immunological parameters were assessed using the Mann–Whitney U test for continuous or ordinal variables, and chi-square or Fisher’s exact test for categorical data. Nonparametric tests were chosen because they are robust to unequal group sizes and do not assume normal data distribution. All statistical comparisons were performed at the group level, and the results were consistent across independent analyses performed for each experimental assay (qPCR, ELISA/Luminex, and flow cytometry; (see **Supplementary Table ST1** for sample distribution).

Fluorescence microscopy images were analyzed using the ImageJ software (version 1.53; National Institutes of Health, Bethesda, MD, USA; RRID: SCR_003070). Quantification performed in Python; ImageJ used for visualization/standardization. Representative images were selected based on clarity and reproducibility.

Overall survival analysis was conducted in patients with PDAC stratified by baseline AHR expression. Kaplan–Meier survival curves were generated, and differences between the Low and High/Medium AHR groups were assessed using the log-rank (Mantel–Cox) test. Survival time was defined as the number of days from blood sampling (typically on or near the day of surgery) to death or last follow-up (June 23, 2025). Statistical significance was set at p < 0.05.

## 3 Results

### 3.1 Modulation of AHR signaling alters target gene expression and inflammatory markers in PDAC-derived PBMCs

**Figure 2:**
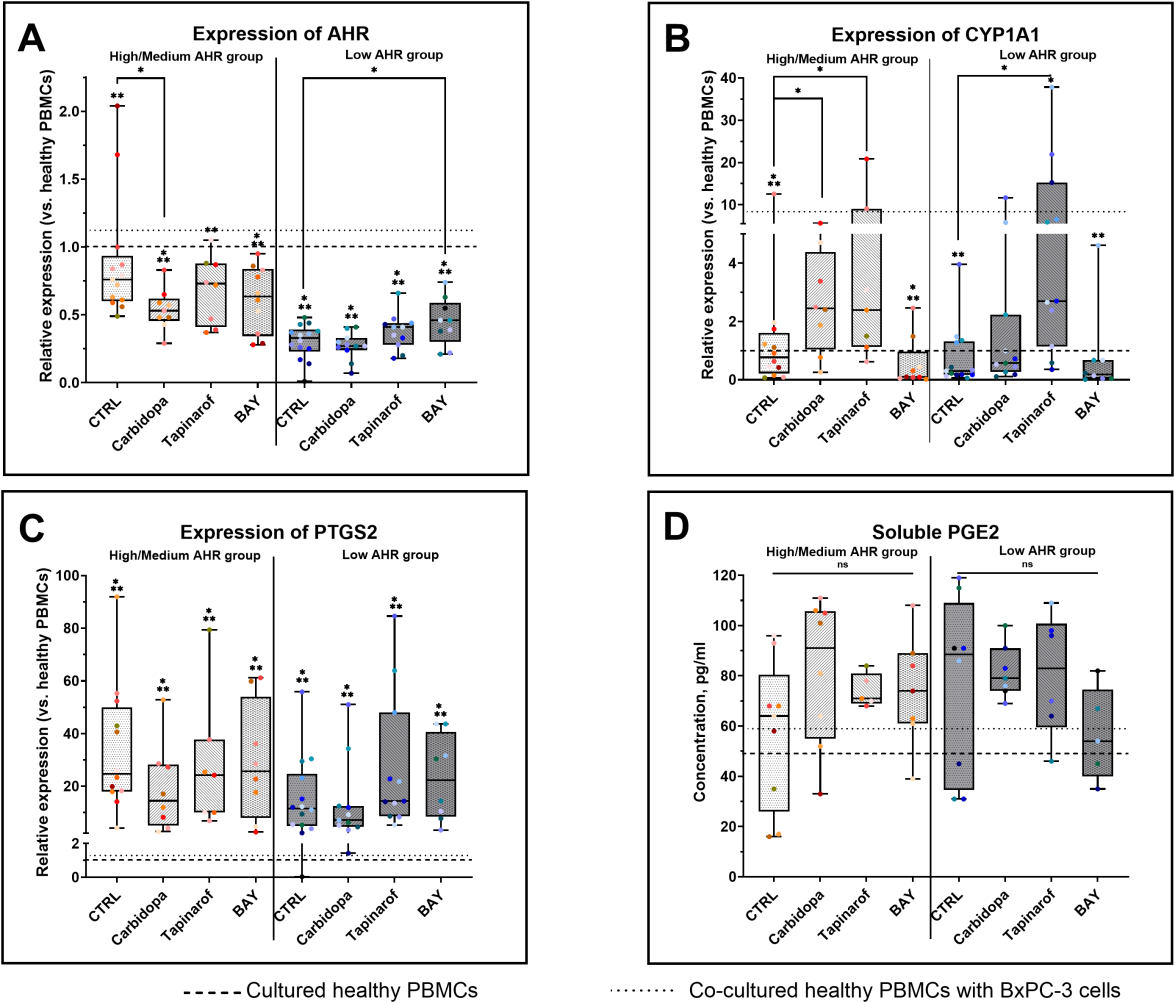
Effects of AHR modulators on gene (qPCR) and protein (ELISA) markers in PDAC-derived PBMCs. AHR gene expression **(A)**, CYP1A1 **(B)**, PTGS2 **(C)**, and PGE2 levels **(D)** were analyzed after treatment with Carbidopa, Tapinarof, or BAY. CTRL refers to untreated PDAC PBMCs stimulated with LPS but without AHR modulators or tumor co-culture. Healthy indicates LPS-stimulated PBMCs from non-cancer donors, and co-cultured refers to healthy PBMCs indirectly co-cultured with BxPC-3 cells. Data shown as medians with full range. Each dot represents one PDAC patient; data from the same individual are shown in the same color across all treatment conditions. Sample numbers (n) varied between assays and AHR expression groups due to differences in PBMC yield and viability (typically n = 5–13 for PDAC High/Medium AHR, n = 5–14 for PDAC Low AHR, and n = 7–14 for healthy donors; see **Supplementary Table ST1** for details). *p < 0.05 vs. healthy; **p < 0.05 vs. co-cultured. ns – not significant.

To evaluate the impact of AHR pathway modulation in PBMCs from PDAC patients, gene expression and inflammatory mediators were assessed across High/Medium and Low AHR expression groups. Treatment with the AHR agonist Carbidopa significantly decreased AHR transcription in the High/Medium AHR group (**Figure 2A**), suggesting receptor repression, whereas the antagonist BAY preserved or slightly increased AHR expression in the Low AHR group in contrast to the reduced baseline. The downstream target gene CYP1A1 was strongly induced by Carbidopa and Tapinarof in both AHR groups, especially at High/Medium AHR, and was suppressed by BAY, indicating effective pathway activation or inhibition (**Figure 2B**). These CYP1A1 changes were used as a pharmacodynamic marker of AHR activation in PBMCs, consistent with previous reports identifying CYP1A1 as a canonical AHR target gene. Compared to healthy PBMCs, PTGS2 expression was markedly elevated in all PDAC groups (**Figure 2C**). Although none of the treatments fully normalized the PTGS2 expression, Carbidopa shifted values slightly closer to the healthy range in both AHR groups. PGE2 levels (**Figure 2D**) remained statistically unchanged across all conditions but showed a modest increase following Carbidopa treatment in the High/Medium AHR group and a decreasing trend after BAY treatment in the Low AHR group, moving marginally toward levels found in cultured healthy PBMCs. These results highlight a differential sensitivity to AHR modulators depending on basal receptor expression and suggest partial normalization of inflammatory signatures, particularly in Low AHR PBMCs treated with BAY.

### 3.2 AHR modulation influences PD-1/PD-L1 pathway

**Figure 3:**
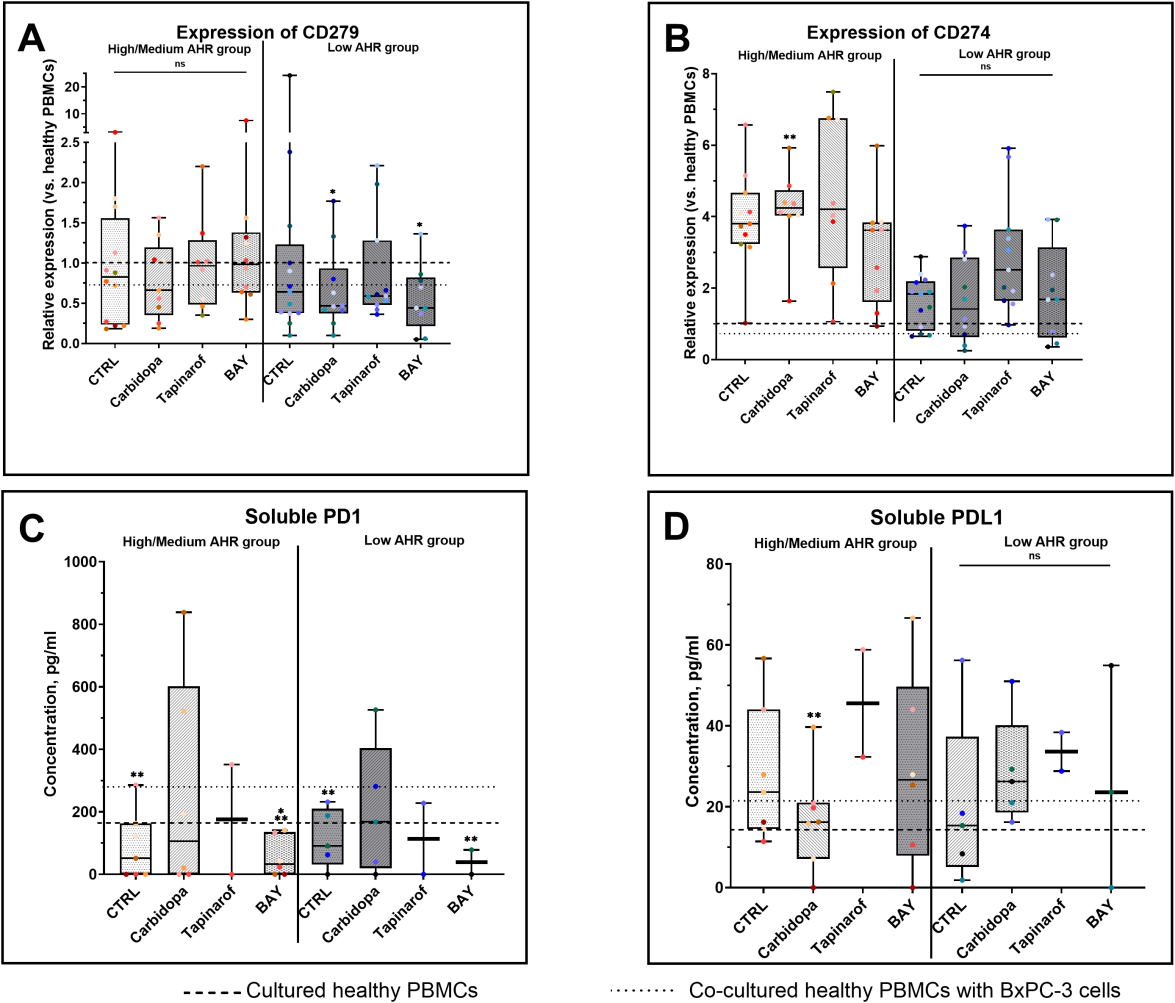
PD-1/PD-L1 gene (qPCR) and protein (ELISA) expression following AHR modulation. CD279 (PD-1) mRNA levels **(A)**, CD274 (PD-L1) mRNA **(B)**, soluble PD-1 **(C)**, and soluble PD-L1 **(D)** were measured in AHR-stratified PDAC PBMCs after modulation. CTRL refers to untreated PDAC PBMCs stimulated with LPS but without AHR modulators or tumor co-culture. Healthy indicates LPS-stimulated PBMCs from non-cancer donors, and co-cultured refers to healthy PBMCs indirectly co-cultured with BxPC-3 cells. Healthy PBMCs and BxPC-3 co-cultured controls included. Data shown as medians with full range. Each dot represents one PDAC patient; data from the same individual are shown in the same color across all treatment conditions. Sample numbers (n) varied between assays and AHR expression groups due to differences in PBMC yield and viability (typically n = 2–12 for PDAC High/Medium AHR, n = 2–13 for PDAC Low AHR, and n = 8–14 for healthy donors; see **Supplementary Table ST1** for details). *p < 0.05 vs. healthy; **p < 0.05 vs. co-cultured. ns – not significant.

In the Low AHR group, both Carbidopa and BAY significantly reduced CD279 (PD-1) expression (**Figure 3A**). In the High/Medium AHR group, Carbidopa treatment only showed a decreasing trend, without reaching statistical significance. No significant changes were observed following Tapinarof or BAY treatment; however, CD279 expression approached the levels observed in healthy PBMCs in Carbidopa treatment conditions, although the response varied depending on baseline AHR expression. Conversely, CD274 (PD-L1) was significantly upregulated by Carbidopa in the High/Medium AHR group, diverging from the healthy baseline (**Figure 3B**). Regarding soluble proteins, Carbidopa treatment increased soluble PD-1 (sPD-1) levels in both AHR expression groups compared with those in untreated PDAC PBMCs (**Figure 3C**), shifting the values closer to those in healthy PBMCs. Although no statistically significant changes were observed in soluble PD-L1 (sPD-L1) levels, a downward trend was noted with Carbidopa treatment in the High/Medium AHR group (**Figure 3D**), which was consistent with the values observed in healthy donor PBMCs. These findings suggest that AHR inhibition via BAY exerts a consistent suppressive effect on PD-1 signaling elements, particularly at the protein level, whereas AHR activation leads to variable transcriptomic effects depending on the baseline AHR expression.

### 3.3 AHR modulation reshapes lymphocyte subset composition in PDAC-derived PBMCs

**Figure 4:**
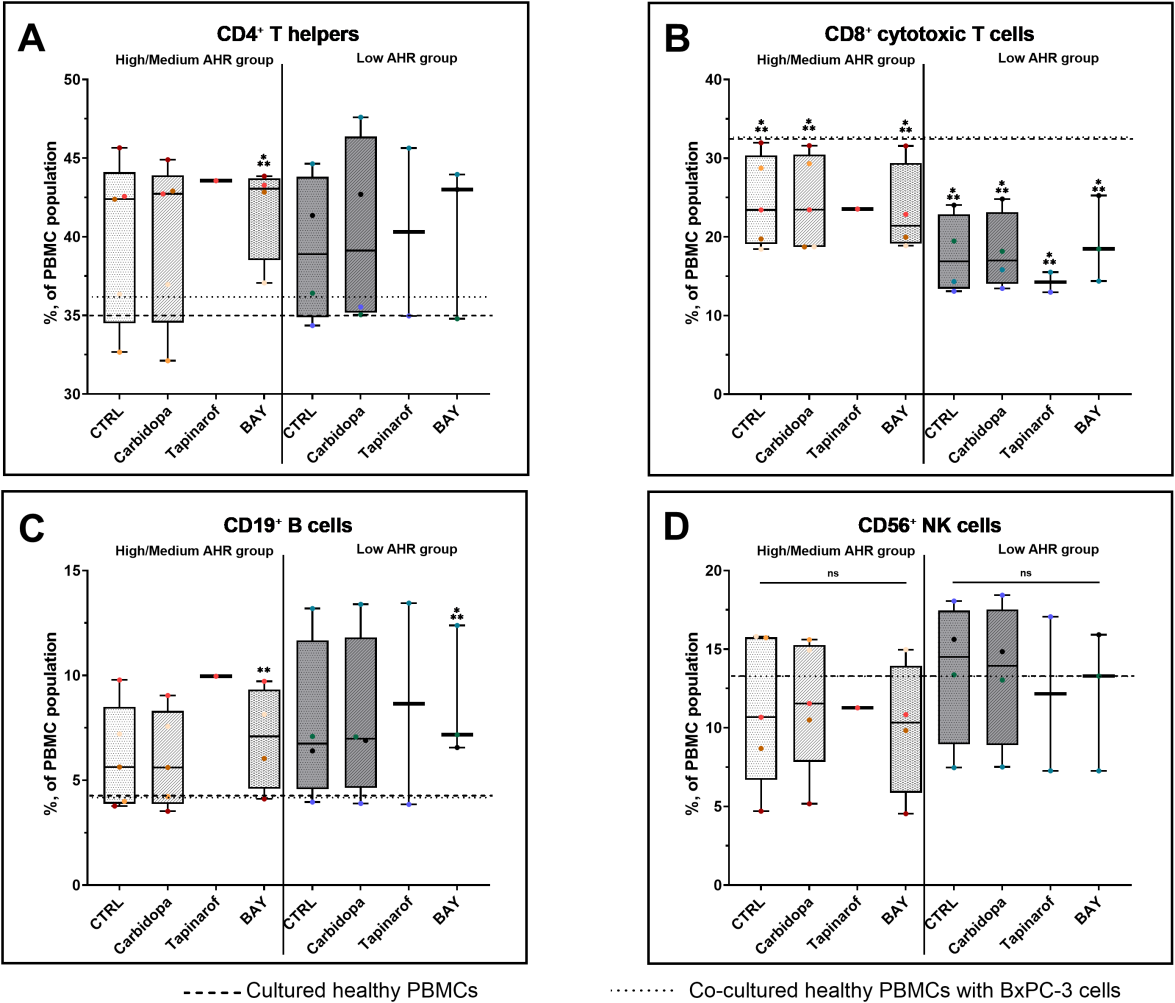
Lymphocyte subset (FC) distribution in PDAC PBMCs after AHR modulation. CD3^+^CD4^+^ T cells **(A)**, CD3^+^CD8^+^ cytotoxic T cells **(B)**, CD19^+^ B cells **(C)**, and CD3^-^CD16^+^CD56^+^ NK cells **(D)** were quantified by flow cytometry. CTRL refers to untreated PDAC PBMCs stimulated with LPS but without AHR modulators or tumor co-culture. Healthy indicates LPS-stimulated PBMCs from non-cancer donors, and co-cultured refers to healthy PBMCs indirectly co-cultured with BxPC-3 cells. Healthy PBMCs and BxPC-3 co-cultured controls included. Data shown as medians with full range. Each dot represents one PDAC patient; data from the same individual are shown in the same color across all treatment conditions. Sample numbers (n) varied between assays and AHR expression groups due to differences in PBMC yield and viability (typically n = 1–5 for PDAC High/Medium AHR, n = 2–4 for PDAC Low AHR, and n = 11 for healthy donors; see **Supplementary Table ST1** for details). *p < 0.05 vs. healthy; **p < 0.05 vs. co-cultured. ns – not significant.

To investigate the immunomodulatory effects of AHR signaling, lymphocyte subsets were analyzed in PBMCs from patients with PDAC stratified by baseline AHR expression. In the High/Medium AHR group, BAY treatment significantly increased the proportion of T helper cells compared with that in healthy PBMCs, whereas no significant changes were observed in the Low AHR group (**Figure 4A**). Cytotoxic T cells had no changes across all treatments in both AHR groups, but their levels were significantly lower compared to healthy PBMCs, especially in the Low AHR group (**Figure 4B**). The percentage of B cells significantly increased following BAY treatment in both AHR groups compared to healthy PBMCs. This increase was part of a broader trend observed in PDAC samples. NK cell frequencies remained unchanged in both groups across all conditions (**Figure 4D**).

### 3.4 AHR modulation differentially affects monocyte polarization in PDAC PBMCs

**Figure 5:**
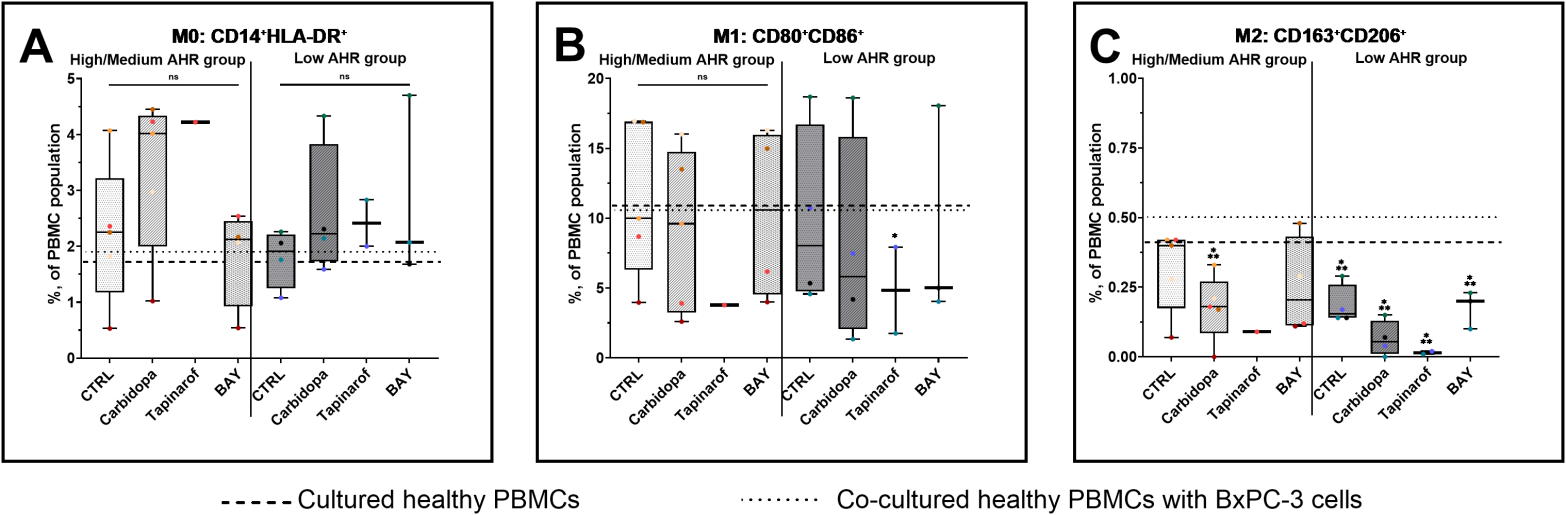
Monocyte (FC) polarization profile following AHR modulation. M0 monocytes **(A)**, M1 monocytes **(B)**, and M2 monocytes **(C)** were assessed in PDAC PBMCs stratified by baseline AHR expression. CTRL refers to untreated PDAC PBMCs stimulated with LPS but without AHR modulators or tumor co-culture. Healthy indicates LPS-stimulated PBMCs from non-cancer donors, and co-cultured refers to healthy PBMCs indirectly co-cultured with BxPC-3 cells. Healthy PBMCs and BxPC-3 co-cultured controls included. Data shown as medians with full range. Each dot represents one PDAC patient; data from the same individual are shown in the same color across all treatment conditions. Sample numbers (n) varied between assays and AHR expression groups due to differences in PBMC yield and viability (typically n = 1–5 for PDAC High/Medium AHR, n = 2–4 for PDAC Low AHR, and n = 11 for healthy donors; see **Supplementary Table ST1** for details). *p < 0.05 vs. healthy; **p < 0.05 vs. co-cultured. ns – not significant.

Analysis of monocyte subpopulations revealed that M0 monocytes remained unchanged across all treatment conditions in both AHR expression groups (**Figure 5A**), with values consistent with those of healthy PBMC and co-cultured baselines, except for Carbidopa, which showed an increasing trend in both patient groups. M1 monocyte levels (**Figure 5B**) were stable in the High/Medium AHR group but significantly decreased after Tapinarof in the Low AHR group, with post-treatment values falling below the healthy reference, indicating a potential suppressive effect on proinflammatory monocytic subsets under Low AHR expression. The most robust and consistent effects were observed in the M2 monocyte subset (**Figure 5C**): all treatments - Carbidopa, Tapinarof, and BAY - led to a statistically significant reduction in M2 cells in both AHR groups. This decrease was especially pronounced compared to both healthy and co-cultured PBMC references, indicating a strong downregulation of this regulatory/anti-inflammatory subset following AHR modulation.

### 3.5 AHR signaling modulates cytokines expression and localization

**Figure 6:**
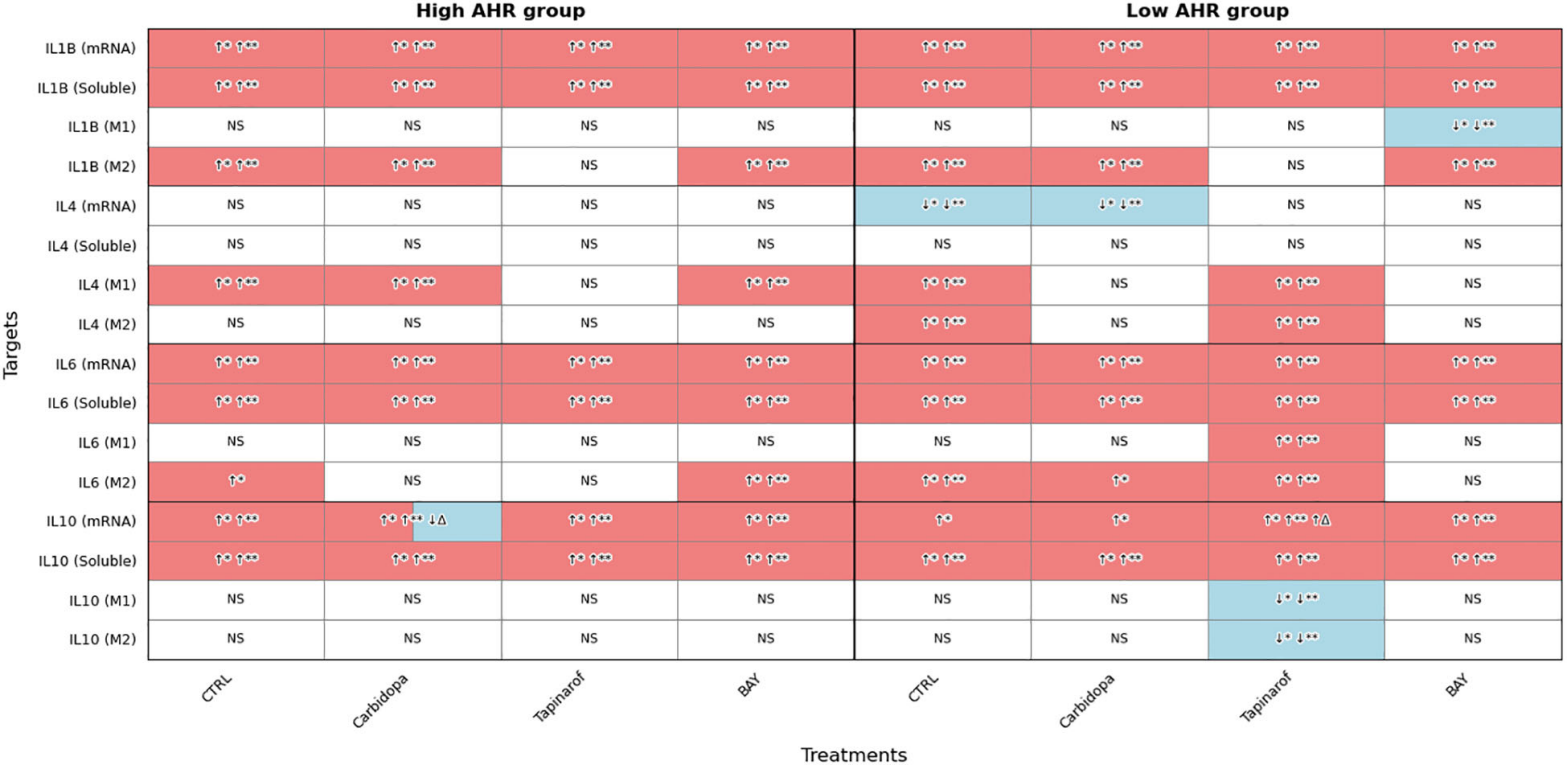
AHR pathway modulation alters IL1B, IL4, IL6, and IL10 expression (FC, Luminex) in PDAC PBMCs. Cytokine expression was assessed at mRNA, soluble, and intracellular levels in M1 and M2 monocytes following treatment with Carbidopa, Tapinarof, or BAY. Samples were stratified by baseline AHR expression. CTRL refers to untreated PDAC PBMCs stimulated with LPS but without AHR modulators or tumor co-culture. Healthy indicates LPS-stimulated PBMCs from non-cancer donors, and co-cultured refers to healthy PBMCs indirectly co-cultured with BxPC-3 cells. Sample numbers (n) varied between assays and AHR expression groups due to differences in PBMC yield and viability (typically n = 1–13 for PDAC High/Medium AHR, n = 2–13 for PDAC Low AHR, and n = 11–14 for healthy donors; see **Supplementary Table ST1** for details). Color coding: red = upregulated; blue = downregulated; white = no significant change; ↑/↓ indicate directional changes; Δ – significant vs. CTRL PDAC; *p < 0.05 vs. healthy; **p < 0.05 vs. co-cultured; NS, not significant.

To investigate the impact of AHR modulation on inflammatory activation, the expression of IL1B, IL4, IL6, and IL10 was evaluated at the transcriptional, soluble, and intracellular levels in PBMCs from PDAC patients stratified by AHR expression level (**Figure 6**).

Transcriptional analysis showed that IL1B mRNA was significantly upregulated across all PDAC groups compared to healthy PBMCs. In parallel, soluble IL1B levels were significantly elevated under all PDAC conditions. Intracellular IL1B in M1 monocytes showed no significant changes in either AHR group, except for a decrease following BAY treatment in the Low AHR group. In contrast, M2 monocytes consistently exhibited elevated IL1B levels across nearly all treatments, except under Tapinarof in both AHR groups, indicating persistent inflammatory signaling within the M2 compartment.

No significant changes in IL4 mRNA were observed in the High AHR group. However, Carbidopa significantly downregulated IL4 mRNA in the Low AHR group. Soluble IL4 levels remained unchanged across all PDAC conditions. Intracellular IL4 in M1 monocytes was upregulated by Carbidopa and BAY in the High AHR group, while in the Low AHR group, only Tapinarof induced IL4 in M1 cells. A similar pattern was observed in M2 monocytes, where Tapinarof significantly increased IL4 expression in the Low AHR group only.

IL6 mRNA was significantly elevated in all PDAC conditions regardless of treatment or AHR expression. Similarly, soluble IL6 levels remained significantly increased, with no observed reduction following any treatment. Intracellular IL6 in M1 monocytes was largely unchanged, except for a notable increase following Tapinarof treatment in the Low AHR group. In M2 monocytes, IL6 remained elevated under BAY treatment in both AHR groups, and also following Carbidopa in the Low AHR group, suggesting a treatment-related modulation of M2-driven inflammatory signaling.

IL10 expression was robustly upregulated at both transcriptional and soluble levels in all PDAC groups. However, only Carbidopa significantly reduced IL10 mRNA levels between treatments in the High AHR group. In the Low AHR group, Tapinarof significantly upregulated IL10 mRNA compared to both healthy and PDAC controls. Similarly, soluble IL10 was elevated in all treated groups. Intracellular IL10 expression remained unchanged in both M1 and M2 monocytes in all groups, except for a significant reduction following Tapinarof treatment in the Low AHR group, suggesting a potential suppression of immunoregulatory signaling under Low AHR conditions.

### 3.6 AHR modulators influence subcellular localization of the aryl hydrocarbon receptor in high/medium AHR group PBMCs

**Figure 7:**
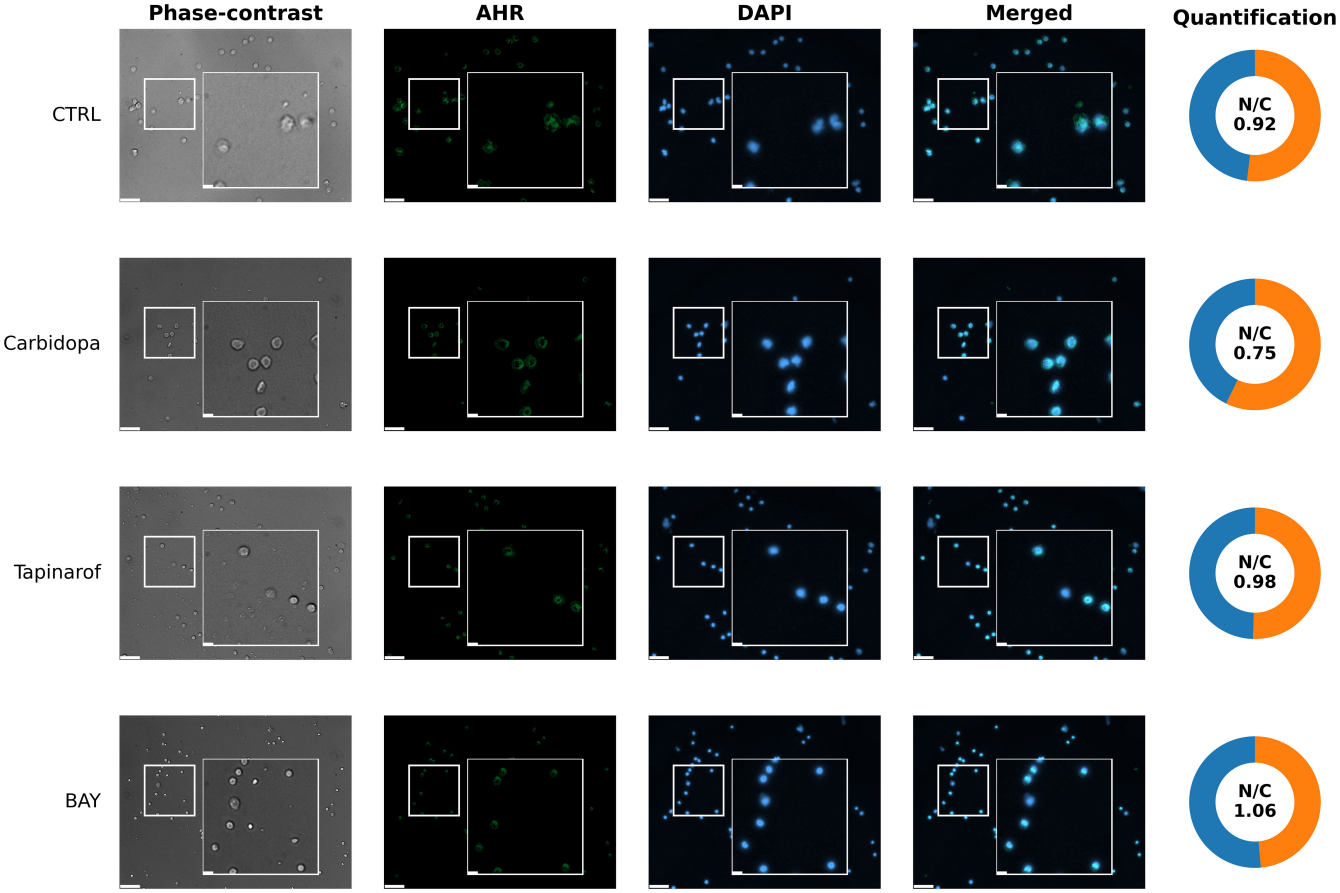
Representative images of aryl hydrocarbon receptor localization (immunocytochemistry) in primary blood cells from patient 256 (High/Medium AHR expression group). Cells were left untreated without modulators (CTRL) or treated with Carbidopa, BAY, or Tapinarof for 24 hours. AHR was visualized using immunofluorescence (green), and nuclei were counterstained with DAPI (blue). Each row corresponds to one treatment condition and displays individual channels (Phase-contrast, AHR, DAPI), merged images, and AHR nuclear/cytoplasmic (N/C) quantification. White squares indicate 3× magnified areas. Scale bar: 50 μm, zoom-in: 10 μm. Representative images are shown for one PDAC donor (patient 256) from the High/Medium AHR group; two biological replicates per group were analyzed in total. Quantitative analysis of both patients is presented in **Supplementary Figure SF5A**.

To assess the spatial distribution of AHR protein in response to pathway modulation, immunofluorescence analysis was performed on PBMCs from a High/Medium AHR group of patients with PDAC (Patient 256). In untreated conditions (**Figure 7**, CTRL row), the aryl hydrocarbon receptor signal was weak and localized predominantly to the cytoplasm, consistent with the inactive receptor status. Exposure to Carbidopa led to a moderate decrease in AHR signal intensity and partial colocalization with nuclear regions (**Figure 7**, Carbidopa row), suggesting ligand-induced receptor activation, translocation and possible autoinhibition. Treatment with Tapinarof resulted in a punctate AHR pattern with a clear nuclear overlap (**Figure 7**, Tapinarof row), indicating enhanced nuclear accumulation. BAY induced the strongest nuclear localization of AHR, with signals sharply concentrated within Hoechst 33342 – stained (DAPI filter) nuclei (**Figure 7**, BAY row), consistent with ligand engagement and nuclear accumulation, which does not imply transcriptional activation. Detailed per-cell quantification (box with jitter) is provided in **Supplementary Figure SF5A**.

### 3.7 Subcellular distribution of AHR protein in PBMCs from a Low AHR PDAC patient following pathway modulation

**Figure 8:**
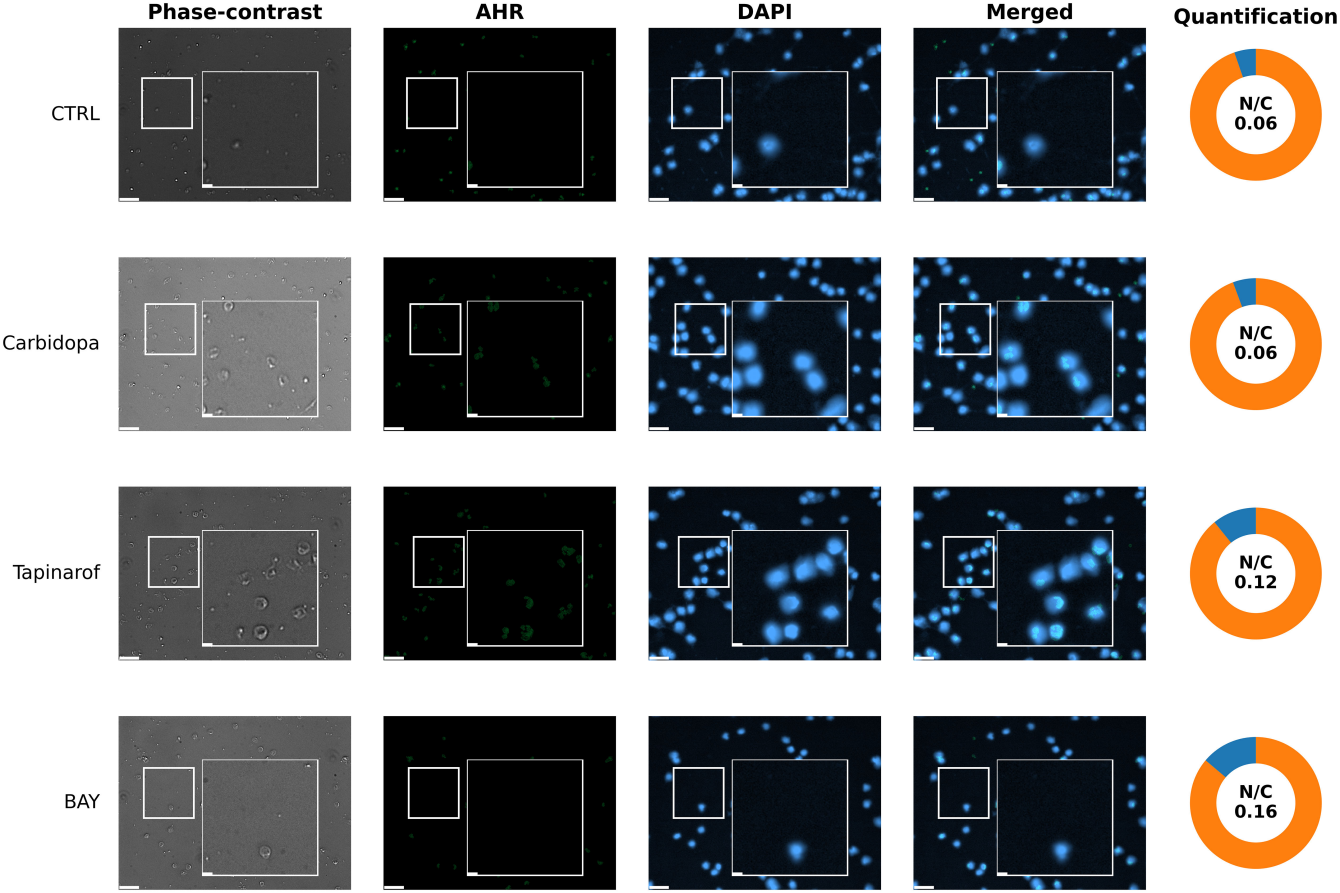
Immunofluorescent visualization of AHR localization (immunocytochemistry) in PBMCs from patient 259 (Low AHR expression group). Fluorescence microscopy images show immunofluorescent labeling of AHR (green) and nuclear staining with DAPI (blue). Cells were either untreated without modulators (CTRL) or treated with Carbidopa, BAY, or Tapinarof for 24 hours. Each row depicts one treatment condition, with corresponding: Phase-contrast, AHR, DAPI, merged images, and donut chart quantification of nuclear and cytoplasmic AHR intensity. White squares indicate 3× magnified areas. Scale bar: 50 µm, zoom-in: 10 μm. Representative images are shown for one PDAC donor (patient 259) from the Low AHR group; two biological replicates per group were analyzed in total. Quantitative analysis of both patients is presented in Supplementary Figure SF5B.

To assess the subcellular distribution of the AHR protein in response to pathway modulation in a patient with low basal AHR expression, immunofluorescence imaging was performed on PBMCs from patient 259. Under untreated control conditions (**Figure 8**, CTRL row), the AHR signal was minimal and dispersed throughout the cytoplasm, with very limited colocalization in the nuclear regions. Carbidopa exposure did not markedly increase AHR signal intensity or alter subcellular localization (**Figure 8**, Carbidopa row), remaining cytoplasmic or diffuse. In contrast, Tapinarof induced a visible increase in AHR fluorescence (**Figure 8**, Tapinarof row), including punctate accumulation within the nuclear areas. BAY treatment (**Figure 8**, BAY row) produced the highest nuclear signal in this patient, although the absolute intensity remained lower than that in the High/Medium AHR patient (**Figure 7**), which is consistent with the overall reduced AHR levels in this group. Quantitative analysis of the nuclear-to-cytoplasmic (N/C) AHR ratio supported these visual findings. Detailed per-cell quantification (box with jitter) is provided in **Supplementary Figure SF5B**.

### 3.8 Clinical association of AHR expression with patient survival

**Figure 9:**
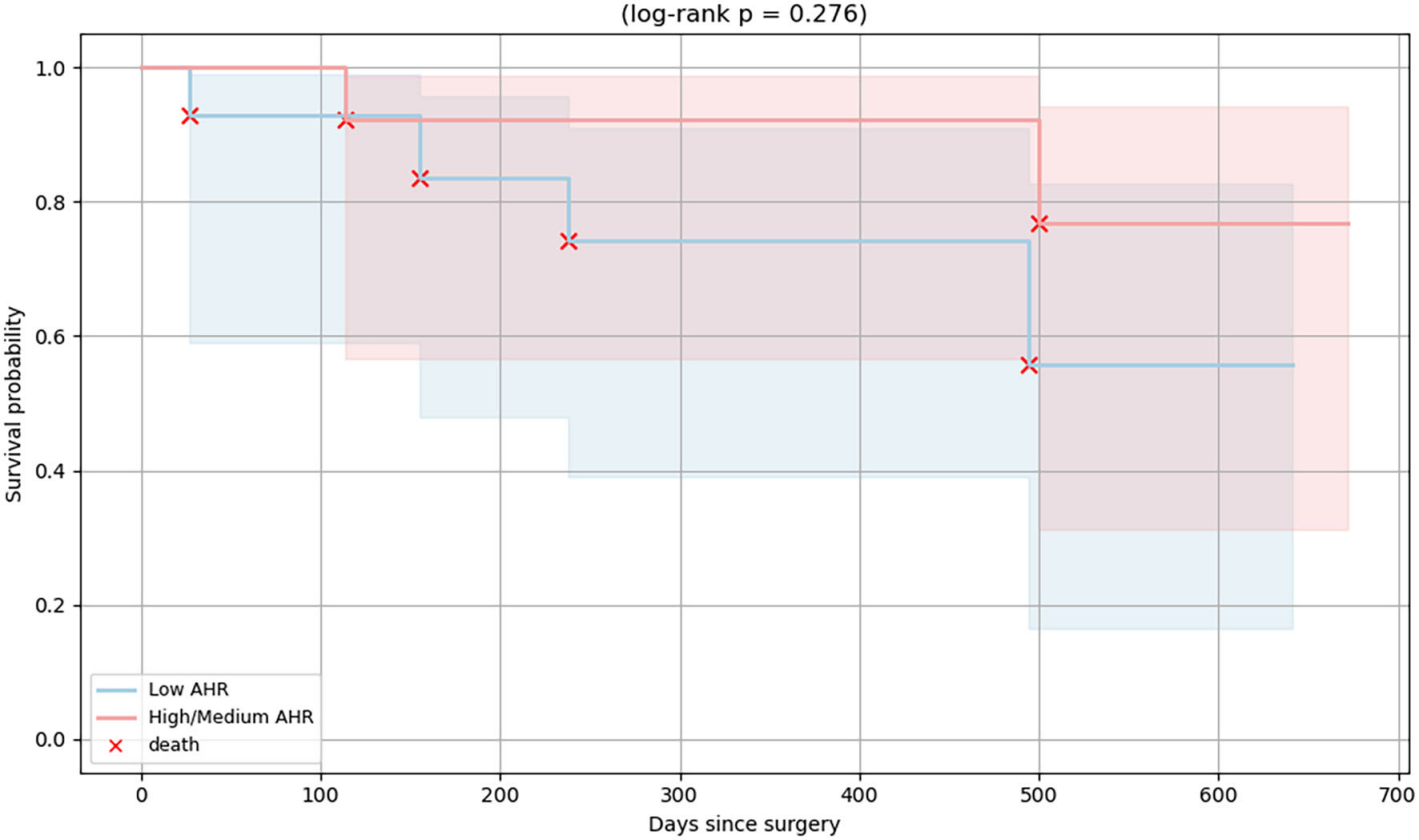
Kaplan–Meier survival curves of PDAC patients stratified by baseline AHR expression. Patients were grouped into High/Medium (red line) and Low (blue line) AHR expression groups. Each red “×” symbol indicates a death event. A non-significant difference in overall survival was observed between the groups (log-rank p = 0.276).

To evaluate the prognostic relevance of baseline AHR expression, overall survival was analyzed in PDAC patients stratified into Low and High/Medium AHR expression groups. Kaplan–Meier survival curves (**Figure 9**) showed a trend toward improved survival in the High/Medium AHR group; however, the difference did not reach statistical significance (log-rank p = 0.276). While exploratory in nature, these results suggest that systemic AHR expression may be associated with immune responsiveness and clinical outcomes, highlighting its potential utility as a prognostic biomarker and tool for therapeutic stratification in PDAC.

To further ensure that the observed immunological differences were not driven by clinical or demographic imbalances, we compared the tumor stage (T, N, M), histological grade (G), age, sex, and serum CA 19–9 levels between the Low and High/Medium AHR expression groups. Using the Mann–Whitney U test for continuous and ordinal variables and chi-square or Fisher’s exact test for categorical variables, no statistically significant differences were observed across groups (all p > 0.05). However, trends toward higher histological grade and more advanced tumor stage (T) were noted in the Low AHR group, although these did not reach statistical significance. These findings suggest that the observed immunological variation is unlikely to result from underlying clinical heterogeneity (see **Supplementary Figure SF1**; **Supplementary Table ST2** for detailed group comparisons).

To further explore potential demographic influences, we examined sex-related immune marker differences in PDAC and healthy donors. An exploratory analysis further compared PD-1, PD-L1, and IL10 expression between male and female PDAC patients (**Supplementary Figure SF2**). A modest increase in PD-1 expression was observed among females within the High/Medium AHR group (p < 0.05), whereas PD-L1 and IL10 levels showed no significant sex-based differences. No such effects were found in healthy PBMCs (**Supplementary Figure SF3**).

## 4 Discussion

The aryl hydrocarbon receptor is a ligand-sensitive transcription factor that plays a key role in the regulation of immune function and tumor development (28, 29). In PDAC, strong changes in AHR activity in both immune and tumor cells contribute to an immunosuppressive environment (19, 27). In our study, Carbidopa unexpectedly lowered AHR gene expression in PBMCs, likely due to negative feedback via AHRR or other self-regulatory mechanisms (30, 31). However, Carbidopa and Tapinarof increased CYP1A1 levels, confirming activation of the AHR pathway. As expected, BAY, a known antagonist, reduced CYP1A1 expression, thus confirming its inhibitory effect (32). In healthy PBMCs, co-culture with BxPC-3 cells increased the AHR and CYP1A1 levels, indicating that signals from cancer cells can activate this pathway (29, 33).

The expression of PTGS2, which drives inflammation and tumor-promoting PGE2 production (25, 34–36), shifted modestly toward the healthy range after AHR agonist treatment (most visibly with Carbidopa) but did not fully normalize, whereas PGE2 changes did not reach statistical significance. These trends suggest that AHR may act differently depending on the context, sometimes promoting or dampening inflammation (26, 37).

In addition to immunological outcomes, we explored whether the clinical features differed between the Low and High/Medium AHR expression groups. While no statistically significant differences were observed, a trend toward improved overall survival was noted in the High/Medium AHR group based on Kaplan–Meier analysis (log-rank p = 0.276). The median survival was not reached in either group during the follow-up period. These findings suggest that baseline AHR expression may have prognostic value in PDAC in addition to its immunological significance. Nonetheless, survival analysis was underpowered and exploratory in nature. As such, no definitive prognostic conclusion can be drawn without validation in larger cohorts.

Similar to Carbidopa, AHR inhibition with BAY reduced PD-1 expression in the PDAC PBMCs. Interestingly, PD-1 expression also decreased in healthy PBMCs after tumor co-culture, suggesting that AHR signaling may be involved in T cell exhaustion (38). Carbidopa showed a trend toward increased levels of sPD-1, a decoy molecule that may help restore immune activity by blocking PD-L1 binding (39). Though its role remains context-dependent, and its elevation has been linked with both immune restoration and poor prognosis depending on disease stage and setting. Thus, interpretation should remain cautious and hypothesis-generating. Simultaneously, the soluble PD-L1 (sPD-L1) levels decreased slightly. A shift in the sPD-1/sPD-L1 ratio may reflect an improved immune responsiveness (40, 41). These results show that AHR-targeting drugs could help fine-tune immune checkpoint activity and potentially complement checkpoint blockade therapy. Importantly, the observed differences between High/Medium and Low AHR PBMC responses highlight the translational potential of AHR expression profiling as a clinical tool to guide the stratified use of AHR-targeted immunotherapies in patients with PDAC.

We also observed changes in lymphocyte populations. BAY treatment significantly increased the proportion of CD4^+^ helper T cells in the High/Medium AHR group, suggesting improved helper cell support, whereas Tapinarof showed only trend-level fluctuations without statistical significance. No significant alterations were observed in cytotoxic CD8^+^ T cells across treatments in either AHR group. The percentage of B cells increased following BAY treatment in both AHR groups, consistent with the overall expansion trend observed in PDAC PBMCs (42). Although we did not directly measure Tregs or Tfh cells, previous studies have shown that AHR can promote Treg development, particularly via kynurenine (43). Our results suggest similar effects, where AHR modulation may tilt immune balance toward enhanced helper and antibody-producing pathways rather than cytotoxic responses. Combining AHR modulators with checkpoint inhibitors may help promote a more effective tumor-killing Th1 profile (44).

Cytokine profiles matched immune cell trends. IL1B, IL6, and IL10 levels were generally increased, with M2 monocytes showing the most consistent upregulation of IL1B and IL6, indicating their major contribution to the inflammatory response. IL1B levels in M1 monocytes remained mostly unchanged, but were significantly reduced after BAY treatment in the Low AHR group. IL6 expression in M1 monocytes also remained stable, except for a marked increase following Tapinarof treatment in the Low AHR group. IL10 was highest in Low AHR samples after Tapinarof treatment at the mRNA and soluble level, possibly indicating regulatory effects. However, intracellular IL10 was significantly reduced in both M1 and M2 monocytes after Tapinarof treatment in the Low AHR group, suggesting that regulatory effects at the transcriptional and secreted levels may not translate into functional immunosuppression within monocytes.

Monocyte polarization supported these findings. The proportion of M0 monocytes remained largely unchanged across treatment conditions, indicating that the observed effects reflect polarization dynamics rather than shifts in total monocyte activation. All modulators reduced the number of M2 cells, which are linked to immune escape. BAY preserved M1 monocytes in Low AHR samples, indicating that AHR inhibition may support proinflammatory and antitumor monocyte profiles (45–48). This observation aligns with the findings of Campesato et al. (2020), who demonstrated that AHR blockade disrupts the kynurenine-driven Treg - macrophage suppressive axis, thereby restoring antitumor immunity and enhancing response to PD-1 blockade. Recent mechanistic studies further confirm the role of AHR in macrophage plasticity within the tumor microenvironment (46). In particular, Abdrabou et al. (2024) demonstrated that inhibition of AHR, together with IRAK1, downregulates the immune checkpoint regulator VISTA and reprograms tumor-associated macrophages toward a proinflammatory, antitumor phenotype (48) supporting the concept that AHR modulation influences macrophage polarization patterns in PBMC-derived monocytes.

An exploratory sex-based analysis based on our data (**Supplementary Figures SF2, SF3**) revealed a moderate increase in PD-1 expression among female PDAC donors within the High/Medium AHR group, whereas no sex differences were detected in healthy controls. This suggests that sex-related immune variation may become apparent only in the PDAC context under heightened AHR signaling. No differences were found for PD-L1 or IL10 in either cohort. Although the sample size is limited, these findings are consistent with recent evidence that AHR signaling interacts with sex hormones and exerts sex-dependent regulatory effects on immune and endocrine pathways (49, 50).Although direct functional assays, such as T cell proliferation, cytokine release (e.g., IFNγ, TNFα), or cytotoxicity measurements, were not performed, the multiplex cytokine profiling and flow cytometric assessment of activation and polarization markers used in this study provide complementary functional insight into AHR-mediated immune modulation. These combined readouts reflect key effector outcomes of AHR signaling, including altered cytokine secretion and immune checkpoint expression, supporting the immunological relevance of the observed AHR-dependent responses.

Subcellular localization data confirmed AHR activity. In High/Medium AHR PBMCs, Tapinarof and BAY led to increased AHR in the nucleus, consistent with activation. In Low AHR cells, this shift was weaker, likely because of lower receptor levels or different activation thresholds. Interestingly, BAY still caused nuclear localization even though it was an antagonist. These imaging data underscore that nuclear localization does not equate to transcriptional activation: despite robust nuclear AHR after BAY 2416964, CYP1A1 was suppressed. This supports the interpretation that BAY 2416964 acts as a selective AHR modulator (SAhRM): it permits AHR nuclear entry but prevents transcriptional activation (31). Other factors, such as AHR nuclear translocator (ARNT) levels or ligand-binding strength, may also affect the extent to which AHR moves to the nucleus.

This study’s findings have several practical implications. Both Carbidopa and Tapinarof are approved drugs with known safety profiles, and BAY is used in clinical testing (31, 44, 51, 52). The AHR-dependent activity of BAY 2416964 and Tapinarof has been experimentally demonstrated in PDAC cell lines by our group (53)where these compounds modulated AHR, PTGS2, and ELAVL1 expression in BxPC-3 and Su.86.86 cells, confirming AHR-pathway specificity. Carbidopa, while not examined in that study, has been independently validated as a selective AHR agonist (51, 52) supporting its use as a pharmacological AHR modulator in PBMC-based assays. Our data support further work on AHR-targeted treatments for PDAC, especially patient selection based on AHR expression, to tailor immunotherapy more effectively.

Moreover, the differential impact of AHR modulators, some of which are already clinically approved for non-oncological indications, raises the possibility of therapeutic repurposing contingent upon further validation in oncology-specific models.

Quantitative AHR profiling of PBMCs could potentially serve as a minimally invasive companion diagnostic tool in future clinical trials, enabling patient selection and real-time monitoring of immune responsiveness during immunotherapy. These findings support the integration of AHR expression profiling into personalized immunotherapy strategies in PDAC.

Direct comparative analysis of AHR expression in TAMs and matched PBMCs has not yet been performed but is currently under investigation. Future work will address whether similar AHR mechanisms occur within the tumor microenvironment and TAMs. Unequal sample sizes across treatment arms and AHR strata arose from variable PBMC yields and *post hoc* stratification. Nevertheless, the use of nonparametric statistics ensures robustness against unequal group sizes, and the observed trends remained consistent across independent experimental readouts (qPCR, ELISA/Luminex, and flow cytometry). The consistency of patterns observed across independent assays (gene expression, cytokine, and flow cytometry analyses) supports the robustness of the overall conclusions despite variable sample sizes.

A limitation of this study is the lack of direct functional immune assays, such as T cell cytotoxicity, proliferation, or cytokine release (e.g., IFNγ, TNFα). Although multiplex cytokine profiling and flow cytometric evaluation of activation and polarization markers provide indirect functional insight, future studies should incorporate these assays to confirm whether the observed molecular and phenotypic changes translate into enhanced immune effector function. Also, the *ex vivo* nature of the PBMC model and use of a single PDAC cell line may not fully reflect the *in vivo* tumor microenvironment. Additionally, several observed trends (e.g., in PGE2, sPD-1) did not reach statistical significance, and survival analysis was exploratory due to small cohort size. Future studies using organoids, functional assays, and prospective cohorts are needed to validate these findings.

## Data Availability

The datasets generated and analyzed during the current study are available from the corresponding author upon reasonable request. All raw data and analysis files supporting the figures are also included in this dataset: Figshare, https://figshare.com/ (DOI: 10.6084/m9.figshare.30636314).
