# Supplementary material for: Baseline AHR expression shapes immune response to pharmacological modulation in PBMCs from pancreatic cancer patients
Source: Front Immunol. 2025 Nov 20;16:1655258. doi: 10.3389/fimmu.2025.1655258 (PMC12675361; doi:10.3389/fimmu.2025.1655258)
Supplement: Supplementary file 1 [file Supplementaryfile1.docx]

**Supplementary Material**


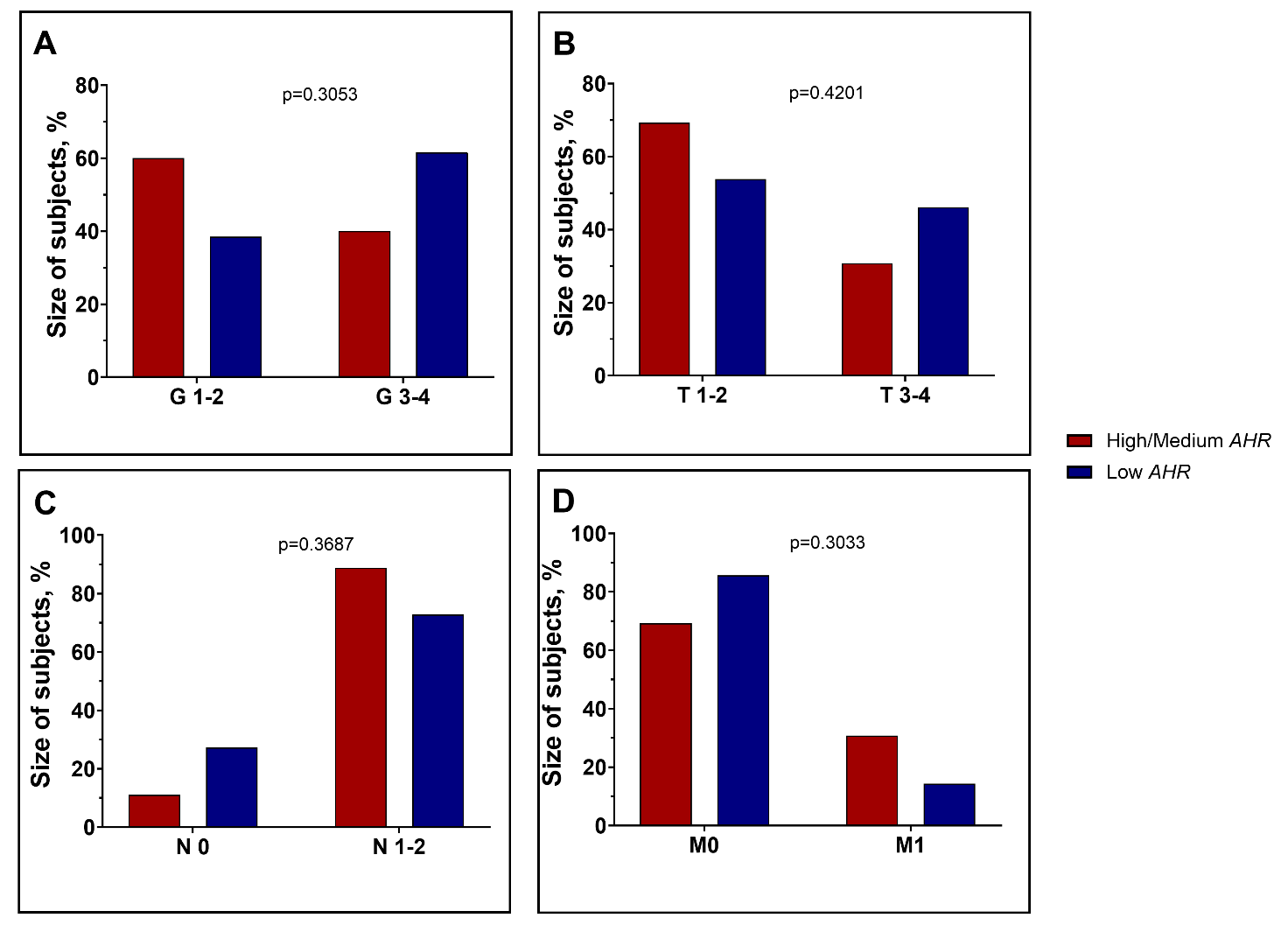


**Supplementary Figure SF1. Clinical characteristics of PDAC patients stratified by AHR expression.**Bar graphs display the distribution of clinical parameters in PDAC patients grouped by AHR expression levels (Low vs. High/Medium).

(A) Tumor grade (G1–2 vs. G3–4).
(B) Tumor size/stage (T1–2 vs. T3–4).
(C) Lymph node involvement (N0 vs. N1–2).
(D) Presence of distant metastasis (M0 vs. M1).

Percentages represent the proportion of patients in each category by AHR expression group. Statistical comparisons were performed using the chi-square (χ²) test. All p-values were >0.05, indicating no statistically significant differences between groups for any clinical parameter. These findings suggest that baseline tumor characteristics were comparable across AHR expression groups. However, trends toward higher tumor grade (G) and more advanced tumor stage (T) were noted in the low AHR expression group.
Note: Data points with missing or undetermined clinical information (indicated as “X”) were excluded from the corresponding analyses.

**Supplementary Table ST1. Number of PDAC and healthy donors per assay, AHR group, and treatment condition.**

Differences in n reflect variable PBMC yields and exclusion of samples failing QC or marker detection thresholds. Complete donor overlap was not required because nonparametric statistics accommodate unequal group sizes.

**Supplementary Table ST2. Demographic and clinical characteristics of PDAC patients by AHR expression group.**

This table summarizes the comparison of baseline demographic and clinical variables between Low and High/Medium AHR expression groups. Age and serum Ca 19-9 values are reported as medians with interquartile ranges (IQR), and sex distribution is presented as counts with corresponding percentages. Statistical comparisons were performed using the Mann–Whitney U test for continuous variables and Fisher’s exact test for categorical variables (sex). No statistically significant differences were observed (all p > 0.05), supporting comparability across groups.


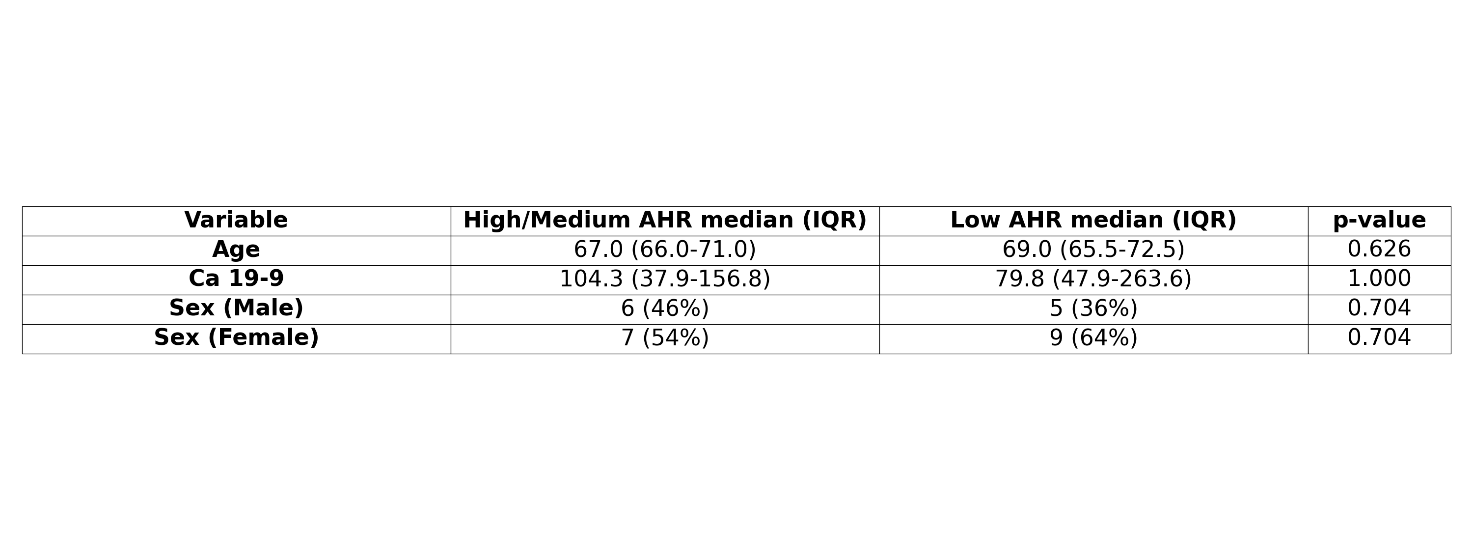


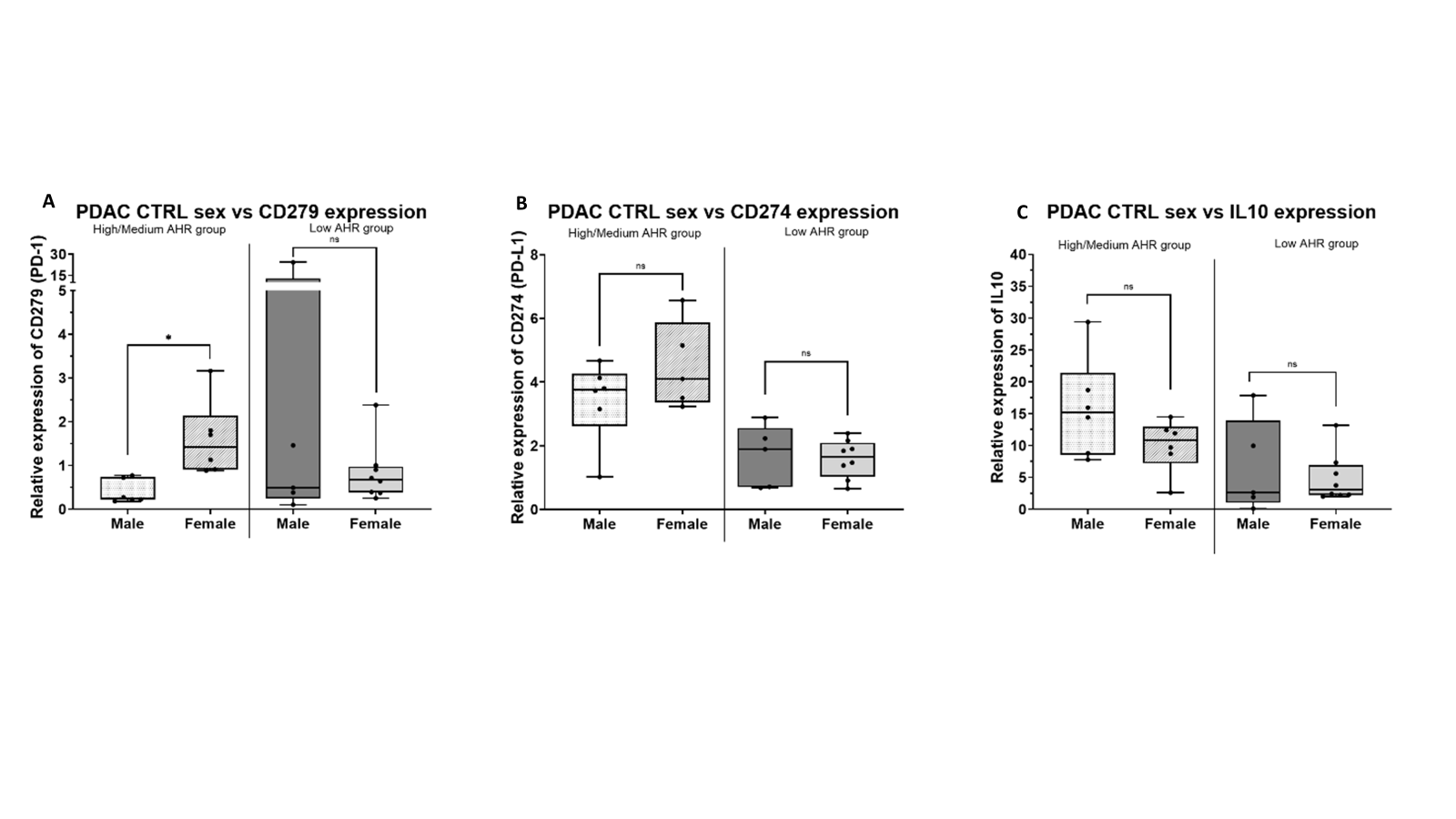


**Supplementary Figure SF2. Sex-based differences in PD-1, PD-L1, and IL10 expression in PBMCs from PDAC patients.** (A) Expression levels of PD-1 (CD279), (B) PD-L1 (CD274), and (C) IL10 were compared between male and female PDAC donors stratified by baseline AHR expression (High/Medium vs. Low). Data are presented as box plots showing median with minimum and maximum values; each dot represents one donor.

A modest but statistically significant increase in PD-1 expression was observed in female patients within the High/Medium AHR group (p < 0.05). No sex-related differences were detected for PD-L1 or IL10 in either AHR subgroup. Statistical analysis: Mann–Whitney U test; p < 0.05 considered significant. These results indicate that sex-based immune variation in PDAC PBMCs may emerge under high systemic AHR activity.


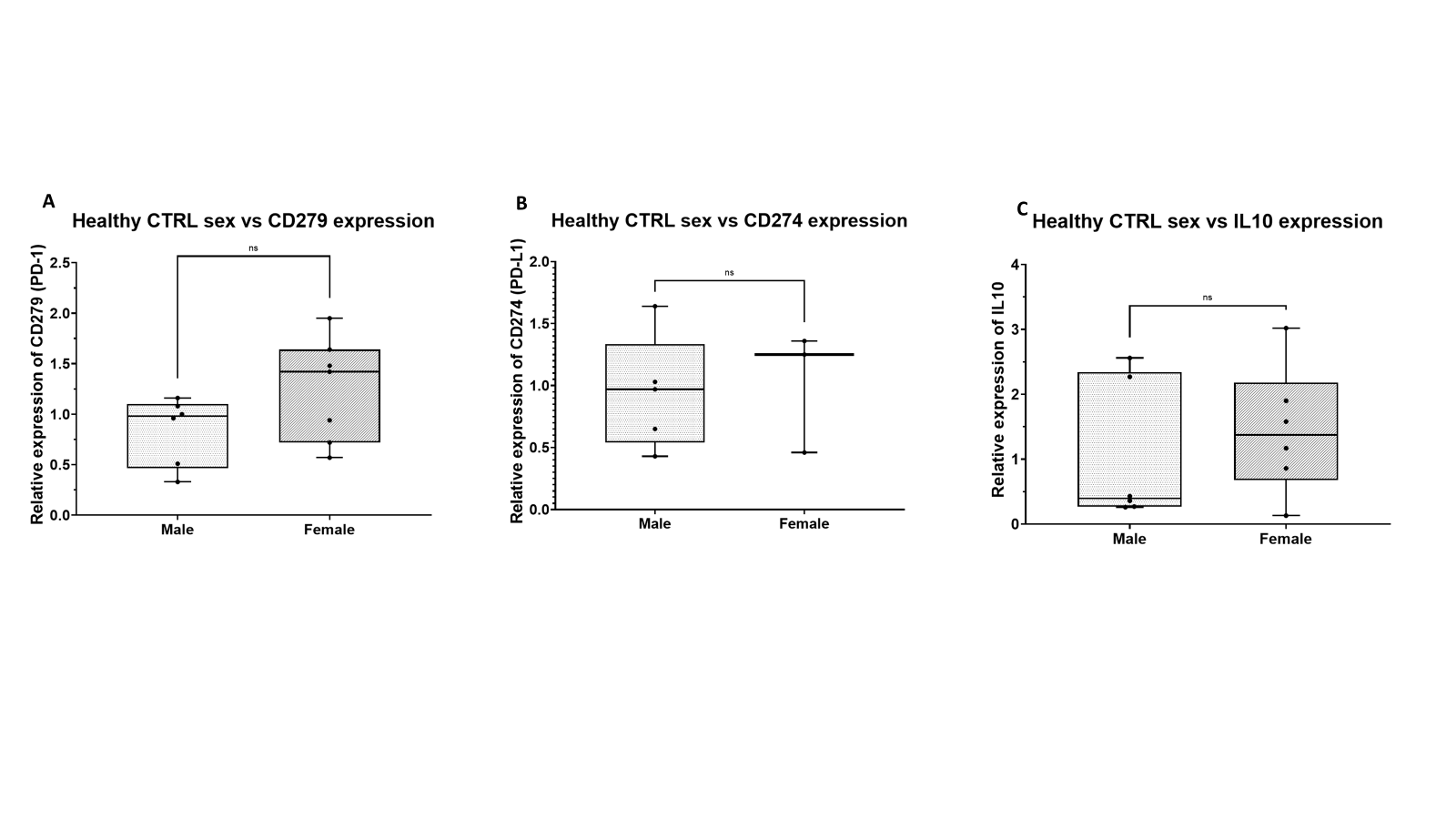


**Supplementary Figure SF3. Sex-based differences in PD-1, PD-L1, and IL10 expression in PBMCs from healthy controls.** (A) expression of *PD-1 (CD279)*, (B) *PD-L1 (CD274)*, and (C) *IL10* was analyzed between male and female healthy PBMC donors under LPS-stimulated control conditions. No statistically significant differences were observed for any of the analyzed markers (all *p* > 0.05). Data are shown as box plots representing median with minimum and maximum values; each dot represents one donor. Statistical analysis: Mann–Whitney U test.


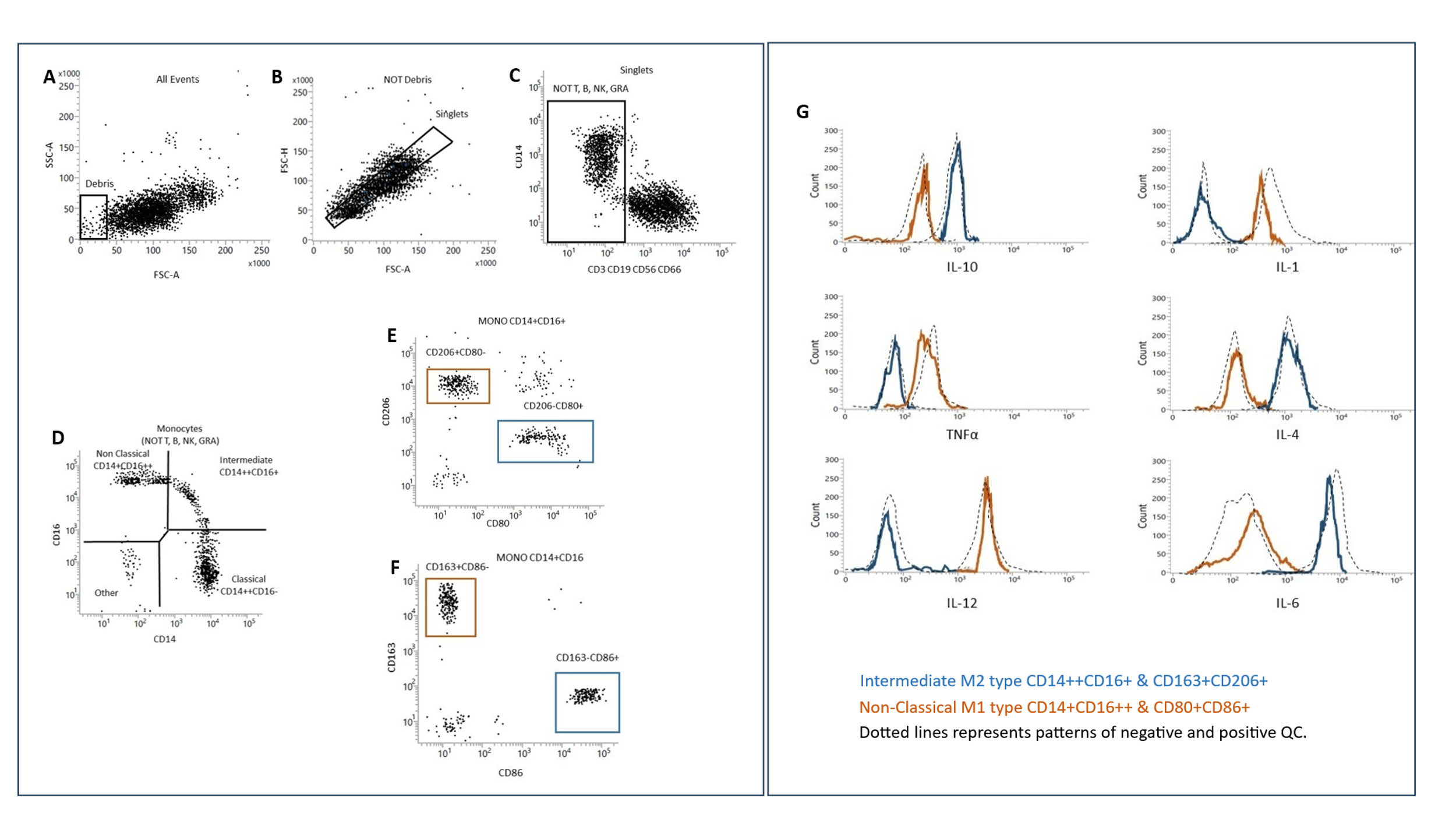


**Supplementary Figure SF4. Flow cytometry gating strategy and quality control.** Representative gating strategy used for PBMC immunophenotyping is shown in panels A–F. Sequential dot plots illustrate stepwise exclusion and identification workflow: (A) all recorded events; (B) debris exclusion based on FSC-A/SSC-A; (C) singlet selection by FSC-H/FSC-A and removal of non-monocytic cells (CD3, CD19, CD56, CD66); (D) monocyte subset discrimination based on CD14 and CD16 expression—classical (CD14⁺⁺CD16⁻), intermediate (CD14⁺⁺CD16⁺), and non-classical (CD14⁺CD16⁺⁺) populations. (E–F) display secondary gating for M1- and M2-like polarization markers within CD14⁺⁺CD16⁺ monocytes. M1-type monocytes were identified as CD14⁺⁺CD16⁺ CD80⁺CD86⁺, and M2-type as CD14⁺⁺CD16⁺ CD163⁺CD206⁺. (G) panels show representative histograms of cytokine expression (IL-1, IL-4, IL-6, IL-10, TNFα, IL-12) for intermediate (blue) and non-classical (orange) monocyte subsets, with dotted lines indicating fluorescence intensity thresholds of negative and positive QC controls.


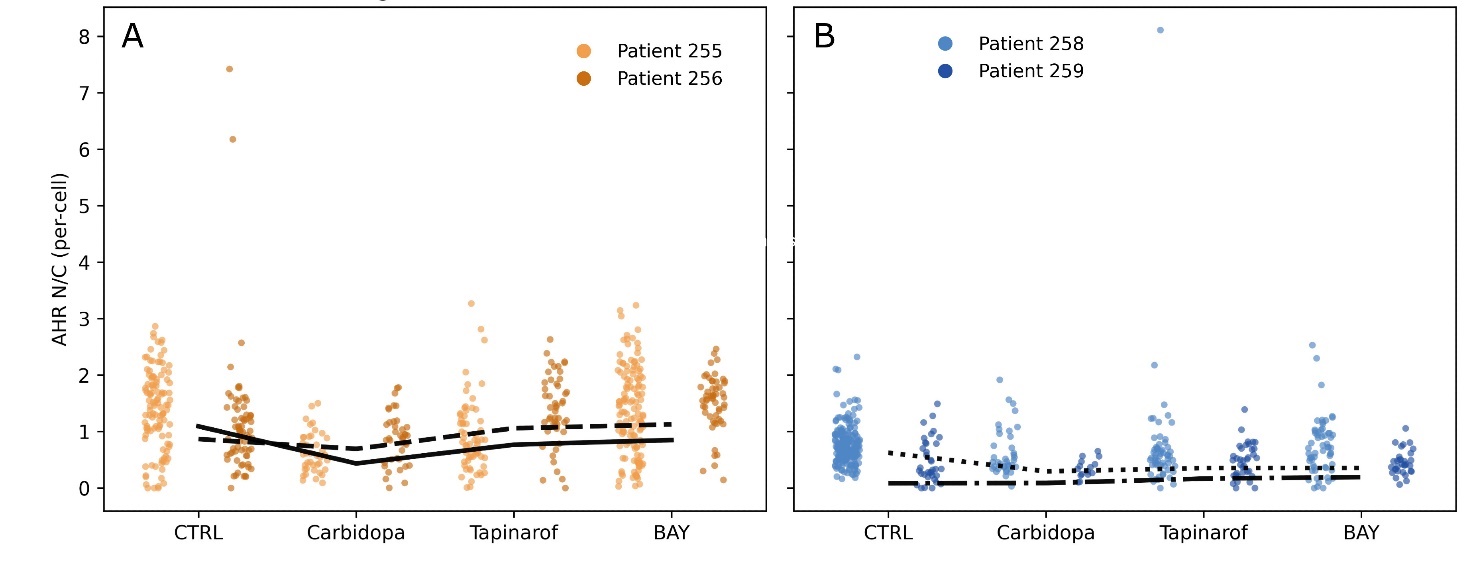


**Supplementary Figure SF5. A - Median AHR N/C localization per patient PBMC cells in High/Medium AHR group (n=2).** **B** - **Median AHR N/C localization per patient PBMC cells in Low AHR group (n=2).** Each point represents a single cell; solid and dashed lines indicate median tendency.


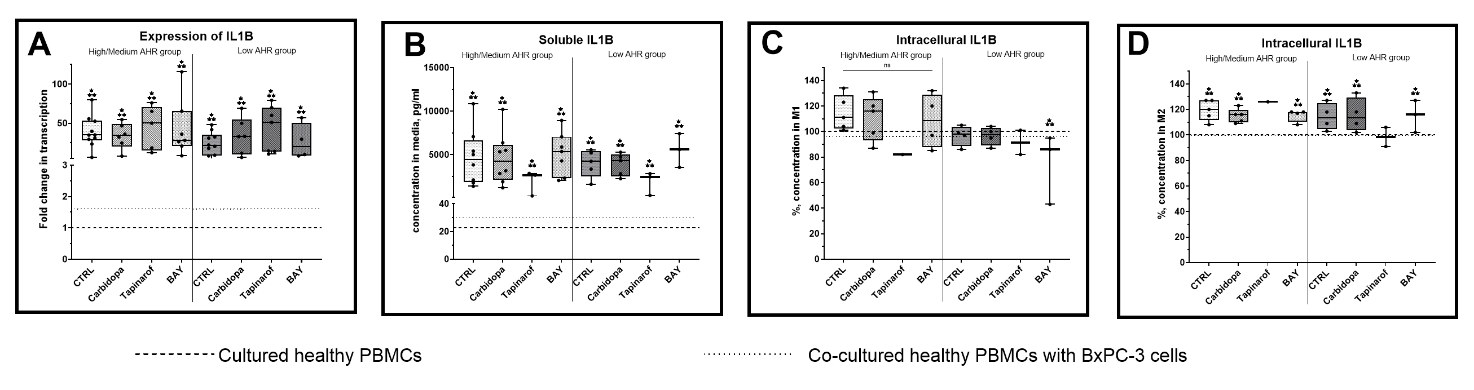


**Supplementary Figure SF6. Separate analysis of IL1B expression at the mRNA, secreted protein, and intracellular protein levels corresponding to Figure 6.** (A) IL1B mRNA expression; (B) soluble IL1B protein concentration (Luminex); (C) intracellular IL1B in M1 monocytes (FC); and (D) intracellular IL1B in M2 monocytes (FC). Samples were stratified by baseline AHR expression (High/Medium vs. Low) and compared with healthy or co-cultured healthy PBMCs. All PDAC and control PBMC samples were LPS-stimulated prior to treatment with AHR modulators (Carbidopa, Tapinarof, BAY). Data are shown as medians with full range, and each dot represents one donor. Dotted lines indicate reference values for cultured or co-cultured healthy PBMCs. *p < 0.05 vs. healthy; **p < 0.05 vs. co-cultured; ns – not significant.


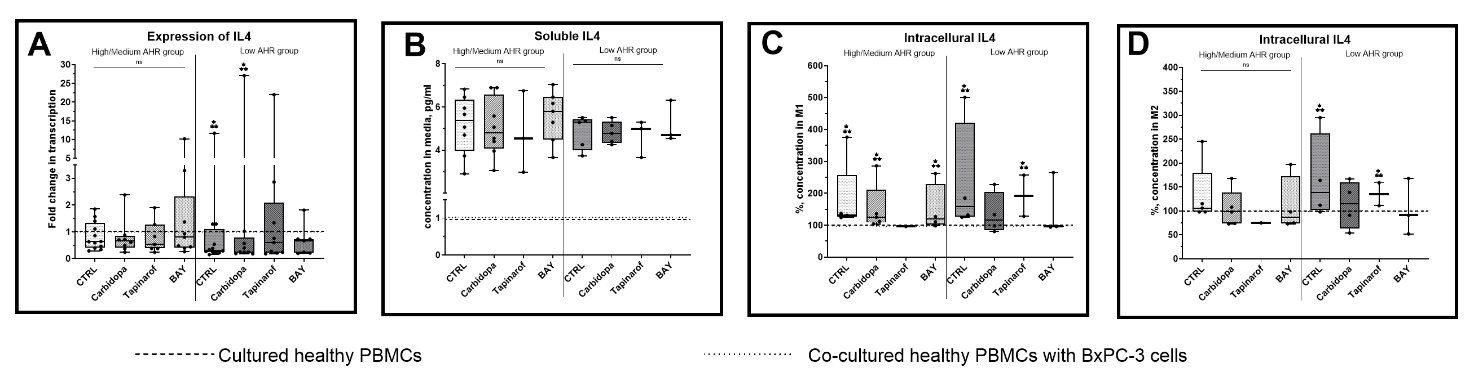


**Supplementary Figure SF7. Separate analysis of IL4 expression at the mRNA, secreted protein, and intracellular protein levels corresponding to Figure 6.** (A) IL4 mRNA expression; (B) soluble IL4 protein concentration (Luminex); (C) intracellular IL4 in M1 monocytes (FC); and (D) intracellular IL4 in M2 monocytes (FC). Samples were stratified by baseline AHR expression (High/Medium vs. Low) and compared with healthy or co-cultured healthy PBMCs. All PDAC and control PBMC samples were LPS-stimulated prior to treatment with AHR modulators (Carbidopa, Tapinarof, BAY). Data are shown as medians with full range, and each dot represents one donor. Dotted lines indicate reference values for cultured or co-cultured healthy PBMCs. *p < 0.05 vs. healthy; **p < 0.05 vs. co-cultured; ns – not significant.


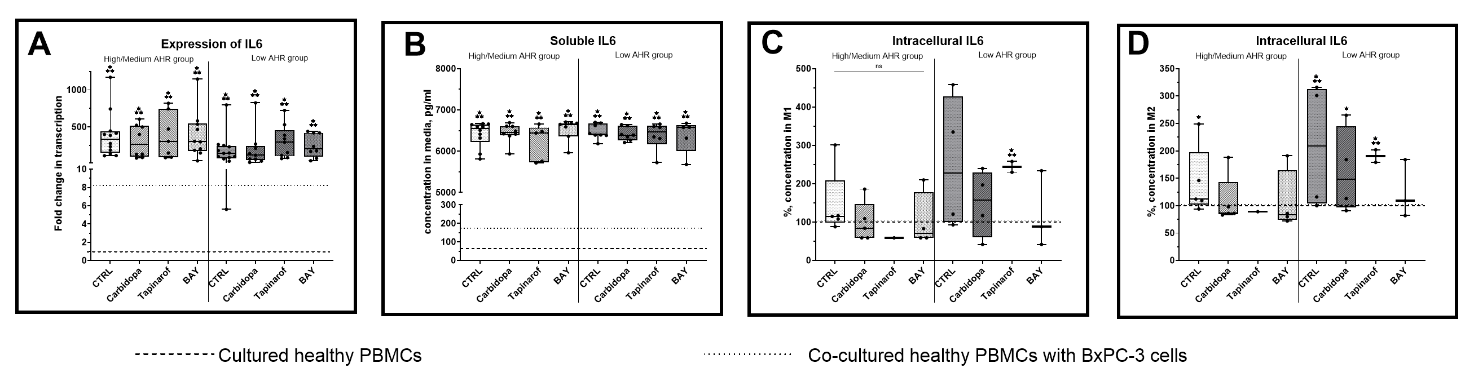


**Supplementary Figure SF8. Separate analysis of IL6 expression at the mRNA, secreted protein, and intracellular protein levels corresponding to Figure 6.** (A) IL6 mRNA expression; (B) soluble IL6 protein concentration (ELISA); (C) intracellular IL6 in M1 monocytes (FC); and (D) intracellular IL6 in M2 monocytes (FC). Samples were stratified by baseline AHR expression (High/Medium vs. Low) and compared with healthy or co-cultured healthy PBMCs. All PDAC and control PBMC samples were LPS-stimulated prior to treatment with AHR modulators (Carbidopa, Tapinarof, BAY). Data are shown as medians with full range, and each dot represents one donor. Dotted lines indicate reference values for cultured or co-cultured healthy PBMCs. *p < 0.05 vs. healthy; **p < 0.05 vs. co-cultured; ns – not significant.


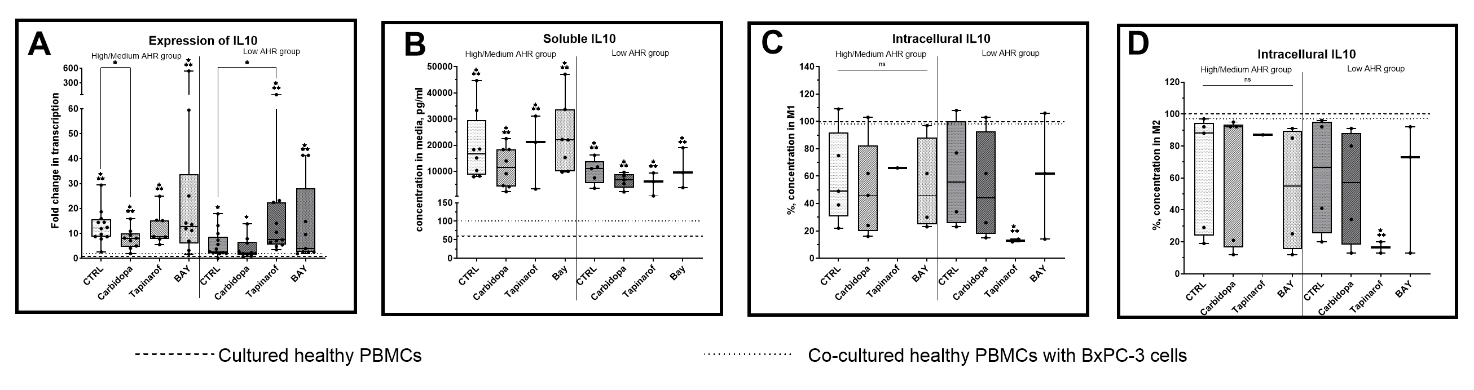


**Supplementary Figure SF9. Separate analysis of IL10 expression at the mRNA, secreted protein, and intracellular protein levels corresponding to Figure 6.** (A) IL10 mRNA expression; (B) soluble IL10 protein concentration (Luminex); (C) intracellular IL10 in M1 monocytes (FC); and (D) intracellular IL10 in M2 monocytes (FC). Samples were stratified by baseline AHR expression (High/Medium vs. Low) and compared with healthy or co-cultured healthy PBMCs. All PDAC and control PBMC samples were LPS-stimulated prior to treatment with AHR modulators (Carbidopa, Tapinarof, BAY). Data are shown as medians with full range, and each dot represents one donor. Dotted lines indicate reference values for cultured or co-cultured healthy PBMCs. *p < 0.05 vs. healthy; **p < 0.05 vs. co-cultured; ns – not significant.
